# Current and future trends in photoacoustic breast imaging

**DOI:** 10.1016/j.pacs.2019.04.004

**Published:** 2019-06-30

**Authors:** Srirang Manohar, Maura Dantuma

**Affiliations:** Biomedical Photonic Imaging (BMPI) & Multi-Modality Medical Imaging (M3I), Technical Medical Centre, University of Twente, PB217, 7500AE Enschede, The Netherlands

**Keywords:** Photoacoustic, Optoacoustic, Breast cancer, Breast imaging, Mammography

## Abstract

Non-invasive detection of breast cancer has been regarded as the holy grail of applications for photoacoustic (optoacoustic) imaging right from the early days of re-discovery of the method. Two-and-a-half decades later we report on the state-of-the-art in photoacoustic breast imaging technology and clinical studies. Even within the single application of breast imaging, we find imagers with various measurement geometries, ultrasound detection characteristics, illumination schemes, and image reconstruction strategies. We first analyze the implications on performance of a few of these design choices in a generic imaging system, before going into detailed descriptions of the imagers. Per imaging system we present highlights of patient studies, which barring a couple are mostly in the nature of technology demonstrations and proof-of-principle studies. We close this work with a discussion on several aspects that may turn out to be crucial for the future clinical translation of the method.

## 1 Introduction

In women, breast cancer is globally the most frequently occurring malignancy, and the leading cause of cancer death. In 2012, 1.7 million women received the diagnosis of breast cancer, 23% of all new cancer cases [1]. In that year about 520,000 women succumbed to the disease, 15% of all female cancer deaths [1]. In future, the incidence and mortality rates in the high Human Development Index (HDI) regions is expected to stabilize, while these will see steep growth in low and medium HDI regions [2]. This is most likely due to adoption of western lifestyles and diets, improvements in life expectancies and increased screening activity.

### 1.1 Imaging for breast cancer detection and diagnosis

Advances in fundamental understanding of breast cancer biology, with steady translation in sophisticated therapies on the one hand, and the dissemination of screening programs on the other, have led to progressively decreasing death rates. Imaging plays a major role in the entire breast cancer management trajectory for detection, diagnosis, neoadjuvant therapy monitoring, guiding biopsies, guiding surgery and for surveillance [3], [4], [5].

Detection of occult breast cancer is the domain of X-ray mammography in screening programs. Diagnosis, following indication of suspicion, is based on clinical examination, X-ray imaging, ultrasound imaging and image-guided needle biopsy. Magnetic resonance imaging (MRI), when available is used in cases of uncertain findings in X-ray imaging and ultrasound imaging [6].

These modalities however suffer from drawbacks. X-ray and ultrasound imaging have non-optimal sensitivity and specificity [5], [7], [8], [9]. Further, X-ray mammography uses ionizing radiation, painful breast compression and has poor performance in radio-dense breasts [5], [10], [11]. For ultrasound imaging, high false positive rates and operator variability in acquiring two dimensional images are limitations [5], [9]. MRI shows high sensitivity by visualizing contrast enhancement in tumor vasculature following bolus injection of Gadolinium contrast [12], [13]. The method has high sensitivity, does not employ ionizing radiation and has good spatial resolution. However, it suffers from limited specificity and requires contrast agents. It is also somewhat logistically hampered by the requirement to time imaging during certain phases of the menstruation cycle in pre-menopausal women [6], [14]. It is also expensive, not universally available, and patients must often be excluded due to claustrophobia, pacemakers, etc. [13].

In view of the impact of breast cancer on society and the shortcoming in the current imaging modalities, there is a continuous search for improved methods for non-invasively imaging the breast and its abnormalities [15]. Several methods and approaches are being intensively investigated for improving sensitivity and/or specificity, as also for improving cost-effectiveness, accessibility, patient burden, personalized care and safety. Examples of promising methods which are being investigated in large-scale clinical trials are digital breast tomosynthesis [16], dual-energy mammography [17], automated whole breast ultrasound [18], ultrasound CT [19], [20], ultrasound elastography [21], diffusion-weighted MR [22], MR elastography [23], MR spectroscopy, breast-specific gamma imaging and positron emission mammography [15].

### 1.2 Tumour angiogenesis and optical absorption contrast

One of the integral hallmarks of cancer has been proposed to be angiogenesis [24], the production of new blood vessels, induced early to support malignant phases in the development of invasive cancers. This process causes locally increased microvascular density with abnormal vessels which are dilated and tortuous [25]. The presence in this enhanced vascularization of hemoglobin (Hb) and its oxygenated variant (HbO_2_), both with strong and specific optical absorption spectra, is expected to provide cancer with an optical absorption contrast with respect to healthy tissue.

It has been demonstrated in several studies using diffuse optical tomography (DOT) with near-infrared (NIR) light [26], [27] that tumours can be visualized based on optical contrast. There is a continuous interest in DOT with advances in source-detector technologies, improvements in modeling, efforts in spatial co-registration or fusion of DOT with X-ray imaging, tomosynthesis, MRI or ultrasound (US), the use of Indocyanine Green (ICG) or other fluorescent contrast agents [27].

However, the biggest impediment to clinical translation of DOT for detection and diagnosis is poor spatial resolution, caused by high light scattering in breast tissue. The contrast is smeared out adversely affecting the detectability of small cancers at early stages of progression. Further, the spatial averaging causes loss of information regarding heterogeneous vascular distribution in the cancer, which can be a further handle in discrimination between malignant and benign lesions, as used in MRI.

### 1.3 Photoacoustic imaging

Photoacoustic (PA), also called optoacoustic imaging [28], can image optical absorption relatively deep in tissue while maintaining high resolutions. The method can visualize blood vessels, and thus the angiogenesis-driven optical absorption contrast of tumours. Light is still the probing energy, but photons are not measured and do not form the detected signal as in DOT. Instead stress (acoustic) waves are detected, which being minimally scattered and attenuated in soft-tissue provide high resolution. The mechanism of photoacoustic signal generation consists of the following steps:

• light is selectively absorbed at higher absorbing regions when tissue is excited by ns pulses of NIR laser radiation,
• the absorbed optical energy *H*(*r*) undergoes fast thermalization,
• the heating produces thermoelastic expansion generating an initial pressure *p*_0_(*r*).

The absorbed optical energy *H*(*r*) is given by the product of the light fluence *ϕ*(*r*) at the absorber and the absorption coefficient (*μ*_*a*_) as:

(1) $$H(r)=\mu_{a}(r)\phi (r)$$

The initial pressure generated *p*_0_(*r*) is proportional to *H*(*r*) as:

(2) $$p_{0}(r)=p(r,t=0)=\Gamma H(r)=\frac{\beta v_{s}^{2}}{C_{p}}H(r),$$

when the laser pulse duration (*τ*_*p*_) is short as to be in the regimes of thermal and stress confinement [29]. In the above, Γ is the Grüneisen coefficient, *β* is the isobaric thermal expansion coefficient, $v_{s}$ is the sound speed, and *C*_*p*_ the isobaric specific heat capacity.

The initial pressure relaxes with the emission of a stress wave, whose propagation is described by an acoustic wave equation [30]. The stress waves have frequencies in the ultrasound (US) range and propagate to the tissue boundary with low scattering and finite velocity. Using a plurality of US detectors, the signals can be detected and the origin of the photoacoustic (PA) sources localized. Thus, while detection of light, would have resulted in washed-out detail due to scattering, detection of US provides high spatial resolution. The PA method combines the rich spectroscopic contrast arising from the use of light as excitation, with the high resolutions arising from low-scattered US propagation and detection. The characteristics of the US detectors determine the resolution of images and imaging depths, and their choices endows a scalability of imaging to the technique not encountered in most other methods. Imaging has been demonstrated at scales of organelles, cells, tissues to whole organs [31]. The characteristics of the light excitation specifically the wavelength, determines the absorbing molecules and thus the biological targets in tissue, as well as light penetration or imaging depths [31]. The distribution and geometrical features of light excitation and US detection on the boundary of the sample can vary considerably, and provide a variety of instrumental implementations depending on the site and application of imaging.

Since the application of NIR-PA for breast imaging was first suggested in 1994 by Oraevsky et al. [32] and Kruger and Liu [33], and first demonstrated in 2001 by Oraevsky et al. [34], many breast imaging prototypes have been reported (see Ref. [35]). True to the flexibility of the PA method, even within the single application of breast imaging, imagers have taken on various measurement geometries, with different choices for US detector materials and characteristics, for illumination schemes and laser source characteristics, and for image reconstruction. In this review, we first analyze the implications on imaging performance of certain design choices of in a generic PA breast imager. We examine clinical PA breast imagers in the literature and present highlights of reported patient studies. We close this review with a discussion on several aspects of the rapidly growing field that are important, and may turn out to be crucial for the future clinical translation of the method.

## 2 Design considerations of a generic photoacoustic breast imager

**Fig. 1:**
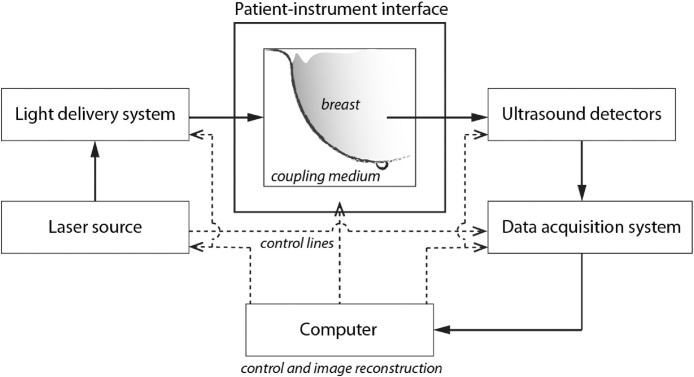
Building blocks of a generic photoacoustic breast imaging system.

A PA breast imaging system comprises the following sub-systems (Fig. 1):

(i) patient-instrument interface,
(ii) ultrasound detector array,
(iii) light delivery system,
(iv) light source,
(v) data acquisition system,
(vi) computer running control and image reconstruction software.

We refer to the coupling of the US detection system with the breast, but also any physical contact of various parts of the imager with the body of the patient, as the patient-instrument interface. This can take various forms and implementations depending on the imaging geometry, and is usually dictated by the geometry of the US detection part. US detectors can have various characteristics which need to carefully chosen as these have a strong influence on the detectability, resolution and imaging depth. The delivery of light to the breast is configured to produce signals in the breast volume within the US detection aperture. The laser is chosen with certain temporal-spatial-spectral characteristics that also have bearing on the detectability of targets and the imaging depth. PA signals produced in a large organ as the breast call for low noise and high dynamic range pre-amplification and digitization electronics. The digitized raw data represent a non-ideal measurement, and are incomplete and noisy, and often with artifacts. The aim of image reconstruction is to recover from this data an accurate high-quality image representation of the breast.

Each building block above has several properties and characteristics which need to be considered in detail and judiciously chosen when developing the imager. These properties and their implications on imager performance will not be addressed explicitly, but are interwoven in the narrative of the paper. We will treat the imaging geometry question explicitly though, since this dictates largely the choices of sub-systems (i), (ii) and (iii) above.

### 2.1 Imaging geometry

The imager configuration depends on the US detection aperture and can be roughly divided into 4 geometries: linear, planar, curved/circular and hemispherical. The geometry has important implications on the performance of the imager. The imaging geometry also largely dictates the design of the patient-instrument interface and the light-delivery system.

*Linear and curvilinear hand-held geometry*. Here hand-held standard linear arrays or custom-developed curved US arrays, operating from US imaging platforms coupled to the laser source are used [36], [37], [38], [39]. The patient can lie supine as in standard breast ultrasonography. Illumination of the breast is on the same side as detection, in the so-called reflection or backward mode. The US array is 2-D focused and light delivery is usually from optical fiber bundles fixed to the array and arranged around it, so as to illuminate the zone coinciding with the 2-D field-of-view of the detector. The US array and light delivery is manually scanned in the region-of-interest in the breast. The US array is acoustically coupled to the tissue using coupling gel or a gel-stand-off pad. In the case of a curvilinear array water or heavy water enclosed within a membrane is used. With this approach, PA imaging leverages on the advantages of US imaging instrumentation namely well-developed hardware and software, compactness, affordability, real-time performance and ubiquity in the clinic [40]. These imagers can be fitted with affordable illumination systems, for example, Refs. [41], [42]. Most imagers are developed as hybrids of PA and US imaging. A dual-mode system provides images of anatomic information from US, overlaid with functional detail from PA imaging. It is relatively easy to combine the two modes as the hardware and software can be shared.

The linear-geometries do come with the usual disadvantages of US B-mode imaging systems namely that only 2-D information is available, and performance is operator dependent. Further, a disadvantage peculiar to PA imaging of the linear array is that of limited-view. Here objects may be inaccurately registered due to boundaries of the object radiating acoustic waves that are not intercepted by the detection aperture [43]. The situation is ameliorated slightly in the curvilinear variant compared to the linear variant, due to the former's greater recording aperture. An example of artifacts produced by limited-view measurements are blood vessels perpendicular to the imaging plane being imaged with only the top and bottom boundaries of the cross-sections visible. Further, reflection-mode PA imaging using 2-D focused linear arrays is susceptible to out-of-plane artifacts [44]. While the artifacts have consequences for image interpretation, missing information from the limited-view is especially a crucial restriction for quantitation of PA images.

*Planar geometry*. The planar geometry uses a 2-D array of unfocused US detectors [45], [46], [47], [48]. The patient-instrument interface can be an examination table or bed, on which the patient lies prone, with the scanner beneath. Access to the breast is via an aperture in the table or bed-top. Alternatively, a patient-upright position is possible as in X-ray mammography, though has not yet been demonstrated. The breast is held against the detector matrix on one side and illuminated from the other side. It should be mentioned that there is fundamentally no requirement for breast compression in PA imaging as applied in X-ray mammography, and breast immobilization is gentle. US coupling gel is applied between the detector and the breast. In certain cases if the field-of-view (FOV) is not large enough, the array is scanned across the breast surface.

The planar geometry has the advantage of a wide detection aperture either synthetically by scanning a small matrix, or physically with the use of a large matrix. A large 3-D volume of the breast can be imaged, if not the whole breast. This makes the geometry less dependent on operator expertise to identify the ROI. Scanning can also be performed under motor control. Further, motion artifacts are minimized since the breast is immobilized. The geometry resembles the X-ray mammography configuration making comparisons and co-registration convenient and relatively straightforward.

A disadvantage in the planar geometry is that while the view is larger than in the linear case, the visibility of object boundaries perpendicular to the 2-D aperture will still be affected though much less than in the linear case. In the planar geometry breast lesions in the proximity of the chest wall are difficult to access. While not a fundamental disadvantage, a drawback can be that 2-D US detector arrays are not available off the shelf as linear arrays. Considerable development work is thus required to make such devices. This also discourages the development of dual-mode imagers, due to the additional complexity in interfacing electronics and switches required to provide US pulsing for US echography.

*Curved and circular geometry*. These imagers use a curved US aperture [49] or a circular (ring-shaped) [50], [51] aperture placed in acoustic contact with the pendant breast, as the patient lies prone. When a plurality of ring-shaped detector arrays along the elevation axis encircle the pendant breast, or if a single ring-shaped US detector array is scanned from chest-wall to nipple, we arrive at the cylindrical aperture geometry. The acoustic coupling can be US coupling gel, water or even dry contact [51]. The US array may be unfocused or focused. Illumination in the curved or circular geometry can be provided using optical fiber bundles [49], or via free-space to illuminate the nipple side of the breast [50]. Another possible strategy with a focused ring-array is to illuminate confocally using an axicon lens as in Ref. [52].

The use of a circular detection aperture encircling the breast provides a complete 2*π* detection angle for the 2-D case. The entire 3-D volume of the breast can be imaged by scanning an appropriate diameter ring-shaped array [52]. The partially overlapping 2-D data sets comprising a synthetic cylindrical aperture can be compounded as in Ref. [53]. While no such systems have as yet been reported, a further advantage is that the geometry lends itself to performing US CT by acquiring data from US transmission/reflection through the breast. By this, co-registered tomograms of sound-speed, acoustic attenuation and reflectivity can be developed as in Ref. [19].

A disadvantage of the use of focused ring-arrays is that information is lost from acoustic field radiated perpendicular or at steep angles to the imaging plane. This makes the recovery of quantitative information from images challenging. Further the in-plane resolution is superior to the slice-thickness. The latter depends on the numerical aperture of acoustic focusing in the plane and is usually quite low; the requirement for focusing to achieve a thin slice demands that the detectors are large along the ring-axis. The net result of both of the above is that accurate representation of anisotropic structures such as blood vessels inclined to the imaging plane, will be affected. A thick ring also means that in its most extreme proximal position, the imaging plane is unlikely to intercept the entire fibroglandular zone which could be detrimental to imaging lesions in the proximity of the chest wall

*Hemispherical geometry*. Here it is sought to perform a full 3-D data acquisition by arranging the US detectors in a hemispherical geometry surrounding the pendant breast when the patient is prone. The detectors can be mounted on the inner surface of a bowl [54], [55], [56], forming a physical aperture, or can take the form of curved arrays arranged in a bowl along the contours of the breast, and scanned to develop a virtual hemispherical aperture [57], [58], [59]. The acoustic coupling in both cases can be water. Some amount of scanning of the detectors is performed, either rotational or linear, to increase the spatial sampling of the imaging volume. Illumination can be provided either from the bottom, or from the sides [56], [57], or both [59].

The hemispherical geometry provides 2*π* steradians of recording aperture. This eliminates the artifacts of limited view seen with other geometries, if the object is enclosed within the detection zone. The amount of information lost from various structures is the lowest of all geometries. The geometry provides the most complete coverage of the breast. The resulting data fidelity is best suited to quantification of images which could enable accurate estimations of blood oxygen saturation (SO_2_) and the metabolic rate of oxygen. Here we have the best possibility of visualizing tumors close to the chest-wall albeit with certain blurred boundaries from where the acoustic wavefront may not intercepted by any detectors.

There are several technical challenges in implementing the hemispherical geometry. A large number of detectors are required, approximately two orders of magnitude larger than in the case of the 2-D circular arrays. The solution is to scan a sparse aperture and synthetically develop a high density aperture with the required number of sampling points. Since the detectors are unfocused and require to interrogate a large volume, they are generally smaller in size than in the focused ring-array. The smaller active area leads to lower sensitivities and SNR, which may require averaging over pulses. The drawback is that averaging and scanning increase the measurement time and the probability for motion artifacts. While in the circular geometry each 2-D slice can be reconstructed separately, for the hemispherical case the entire 3-D data set is used. This amounts to a large computational burden that can discourage the use of sophisticated iterative and model-based image reconstruction algorithms as can be applied in the other geometries. Real-time imaging with high resolutions can be difficult to implement with volumetric image reconstructions. There is however progress is developing strategies in the framework of compressed sensing theory that may permit real-time imaging from reduced data sets. This exploits the fact that photoacoustic absorbers in tissues are sparse yielding redundancy in regular Nyquist sampling. Using only a sub-set of detectors points chosen in a random fashion to maximize non-redundancy, it has been shown possible to reconstruct images from incomplete datasets [60], [61]. Using this approach, with a lower number of detector points than recommended by the Nyquist criterion, real-time imaging in the hemispherical geometry could be possible with acceptably high resolutions.

## 3 Clinical breast imagers and imaging results

**Fig. 2:**
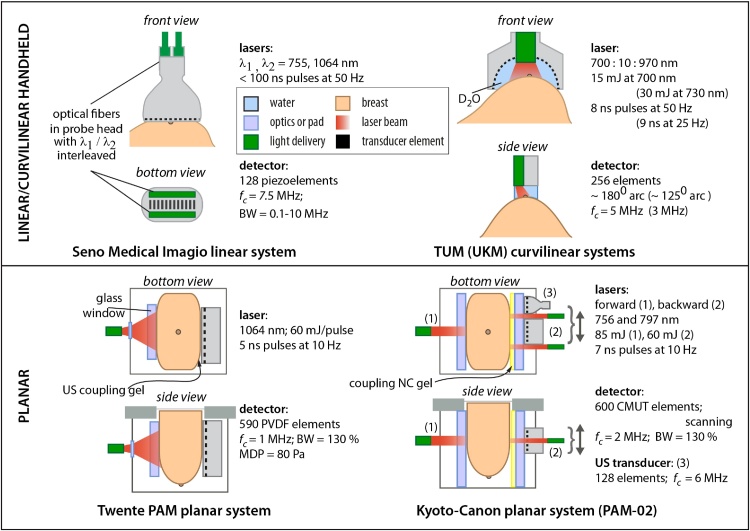
Schematic drawings of photoacoustic breast imaging instruments with linear, curvilinear and planar detection apertures, with selection of details of their lasers and ultrasound detectors. Acronyms – TUM: Technical University of Munich; UKM: University Hospital Münster (see also Fig. 10).

A handful of PA breast imaging systems have been applied in patient studies. Here we describe the technical details of these in turn, reproducing their representative breast images and summarizing their patient results. Figs. 2 and 10 consolidates the configurations of the imagers showing their imaging geometries, with details of light sources, detectors and coupling media.

We henceforth refer to the imagers by the name of the company or University that has developed the systems, or performed the clinical studies.

### 3.1 The Seno Medical Imagio linear system

This device has been developed by Seno Medical Instruments (San Antonio, TX, USA) and seeks to improve breast cancer diagnosis, combining PA with US to potentially provide a better discrimination between malignant and benign masses. The Seno Imagio™ breast imaging system, which received a CE mark in April 2014, is intended for investigational use in the United States until Pre-Market Approval is finalized by the FDA.

#### 3.1.1 Instrumentation

Technical details of the system are to be found in Ref. [62]. The hand-held linear array was used as a stand-alone grayscale US transducer and as a PA detector. The US component was designed to meet specifications comparable to state-of-the-art US machines and transducers in current clinical use [63]. The device can be set to US mode, generating only real-time grayscale US images. When in PA/US mode, grayscale US images are interleaved with fused functional PA data for real-time display. When in PA/US mode, the received PA signal is color-coded for relative oxygenation (green to aqua going from most oxygenated to least oxygenated) and deoxygenation (red to pink for descending deoxygenation) of hemoglobin [63].

*The Ultrasound Detector and light delivery*. The linear array has 128 elements in a 38.4 mm linear array with 7.5 MHz center frequency. System bandwidth is 0.1–10 MHz at −6 dB level [62], [64].

Two lasers are used interleaved, an Alexandrite (755 nm) and an Nd:YAG (1064 nm). The laser pulse duration is <100 ns, with optical fibers transmitting the energy to both sides of the probe. Light is diffused across the surface area of the light bars on either side of the linear array transducer [62]. The energy is calibrated to a reference pyroelectric sensor to ensure the lasers are producing equivalent light output, with a homogeneous density. Radiant exposure is maintained at 20 mJ cm^−2^ for both wavelengths.

*Signal Processing and analysis*. The reconstruction is a filtered backprojection method with post-processing to enhance differentiation between the two laser wavelength images [64].

#### 3.1.2 Patient studies

The results of two clinical studies have recently been published – the Pioneer study in the United States, and the Maestro study in the Netherlands.

*Pioneer: pivotal study of the Imagio breast imaging system*. The study goal was to ascertain the diagnostic utility of PA/US images (PA and gray-scale US) compared with conventional US images in differentiating benign and malignant breast masses [63]. The study was a prospective, multi-center study at 16 sites in the USA involving 2105 women, easily the largest using the PA method till date [63].

Masses designated from diagnostic US as BI-RADS (Breast Imaging Reporting and Data System) 3, 4, or 5 with biopsy-proven histologic findings, and masses designated as BI-RADS 3 stable after 1 year, were eligible. PA/US imaging was performed by trained site investigators. One imaging protocol was applied at all sites: standard orthogonal images in both the US and PA/US modes were acquired. Orthogonal video loops in both modes were also acquired to simulate real-time scanning for independent readers.

Seven dedicated and experienced breast radiologists served as readers. A training study was performed with an initial cohort of 100 subjects. In the subsequent study, the readers were blinded from all subject data having access only to PA/US imaging. Independently, the readers assigned BI-RADS categories and a probability of malignancy (POM) for the US-alone images first. After these results were locked, the readers reviewed PA/US images, scored PA features, and assigned PA/US POM and a BI-RADS category. The PA features and the scoring criteria are reported comprehensively [63], and will not be repeated here. Histologic assessment was based on hematoxylin–eosin (H&E) stains, and details of histologic grading and additional lesion attributes extracted may be found in Ref. [63].

Following exclusions due to technical malfunction, protocol deviations, etc., the final reader population comprised 1690 subjects (1757 masses). The following are the most important findings:

(1) *Absolute specificity advantage and sensitivity*

PA/US had an advantage of about 15% over US. The sensitivity of PA/US (96%) was non-inferior to that of US (98.6%).

(2) *Downgrading BI-RADS in benign masses*

**Fig. 3:**
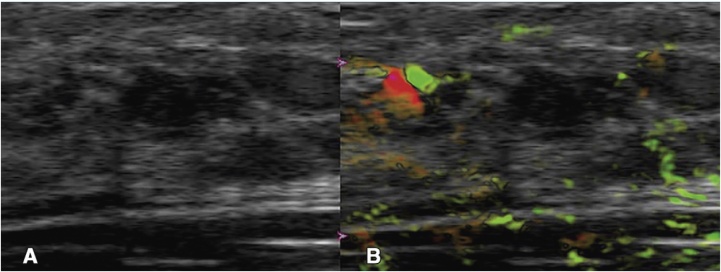
From the Pioneer study using the Seno Imagio linear system: example of downgrading BI-RADS 4A to 3. Images in a 25-year-old woman: (A) internal US image showing mass assessed as a BI-RADS 4A with recommendation for biopsy. (B) PA/US combined map with no internal and external OA features, resulting in a low OA score and a downgrade to BI-RADS 3. Biopsy revealed benign fibrocystic changes. (Reproduced with permission of the authors and publishers.)

PA/US resulted in downgrading 34.5% of benign masses (from BI-RADS 4A or higher to BI-RADS 3 or 2, or from BIRADS 3 to BI-RADS 2). An example is shown in Fig. 3, where the US image in a 25-year old woman visualized a hypoechoic mass with indistinct and angular margins assessed as a BI-RADS 4A by 5 of 7 readers with recommendation for biopsy. The PA/US combined map showed a complete absence of both internal and external PA features, resulting in a low PA score and a downgrade to BI-RADS 3 by 4 of 5 readers. Biopsy revealed benign fibrocystic changes. In such a case, PA/US could have potentially avoided an unnecessary biopsy.

(3) *Upgrading BI-RADS in Malignant Masses*

**Fig. 4:**
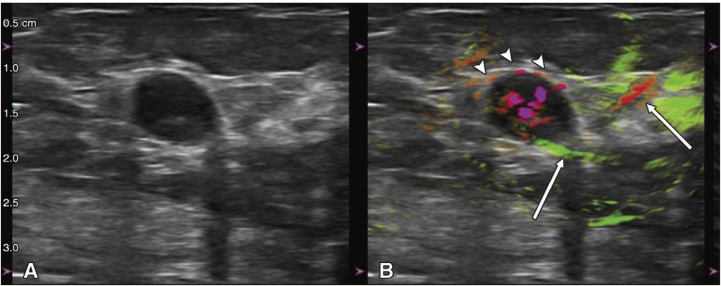
From the Pioneer study using the Seno Imagio linear system: example of upgrading BI-RADS 3 to 4A or higher. Images in a 71-year-old woman: (A) internal US image showing mass assessed as BI-RADS 3. (B) PA/US combined map reveals abundant intensely deoxy-Hb within the tumor interior, deoxy-Hb blush within the echogenic rim that represents the tumor boundary zone (arrowheads) and radiating peripheral artery (green) and vein (red) (arrows). BI-RADS 3 assessment was upgraded to 4A or 4B. Biopsy revealed triple-negative IDC. (Reproduced with permission of the authors and publishers.)

PA/US resulted in upgrading 47.0% of malignant masses classified as BI-RADS 3 with US to BI-RADS 4A or higher. Fig. 4 shows an example. The US image of the 71-year old subject shows a mass assessed as BI-RADS 3 by 3 of 7 independent readers. The PA/US combined map shows deoxy-Hb in the tumor interior, deoxy-Hb blush at the tumor boundary zone (arrowheads) and a radiating peripheral artery (green) and vein (red) (arrows). Based on these features, readers upgraded assessment to BI-RADS 4A or BI-RADS 4B. Biopsy revealed triple-negative IDC. In such a case, in the clinic, the addition of PA/US would have increased diagnostic confidence to recommend biopsy.

(4) *Diagnostic performance*

Specifically, the addition of PA compared with US alone showed potential for achieving higher specificity in assessment of benign and malignant breast masses. This can potentially reduce the number of false-positive examinations and unnecessary biopsies of benign masses. In general, the diagnostic performance of PA/US compared favorably with other functional breast imaging modalities and techniques as contrast-enhanced MR imaging, PET, scintimammography, color and power Doppler, and strain and shearwave elastography.

*Maestro: Imaging with opto-acoustics to downgrade BI-RADS classification*. The aim of the Dutch study was focused on assessing the ability of PA/US to assist in downgrading benign masses classified as BI-RADS 4A and 4B to BI-RADS 3 or 2 [65]. This was a prospective, controlled, and multi-center study at five centers with an inclusion of 209 patients.

Masses classified as BI-RADS 4A or 4B based on clinical US imaging were included. A PA/US examination was performed after conventional US and before biopsy. Standard histopathology was performed on biopsied tissues and underwent a central pathologic review by an independent pathologist. This was considered as the reference standard for PA/US comparison. The PA/US results were interpreted and evaluated in a non-blinded manner with access to all participant data and background clinical information as in a real-world clinical situation. The investigators were dedicated breast radiologists with a minimum of 5 years of experience, and underwent formal training in performance and interpretation of PA/US. Five PA/US features were scored and a probability of malignancy (POM) on a scale from 0% to 100% was estimated. Based on the PA/US findings, when appropriate, US-assigned BI-RADS classification were adjusted.

Following exclusions due to technical failures, protocol deviations, etc., the final intention-to-diagnose population comprised 209 subjects (215 masses). The following are the most important findings:

(1) *Downgrading BI-RADS in benign masses*

Of 146 benign lesions, 60 were correctly downgraded from BI-RADS 4A or 4B to BI-RADS 3 or 2 using PA/US examinations. Of the benign masses designated as BI-RADS 4A (119), 48% were downgraded to BI-RADS 3 or BIRADS 2. The implication of this is that addition of PA/US examinations could reduce the number of biopsies which are negative for cancer, and the necessity for short interval follow-up imaging examinations.

(2) *High accuracy of PA/US findings in malignancies*

A low rate of false-negative findings (4.5%) was reported, with only 3 numbers of false-negatives from 67. The true-positive rate was thus 95.5%. The three false-negative readings were attributed to investigators’ lack of experience in interpretation since the findings were in the first 50 inclusions [65]. None of the false-negative lesions were due to inadequate light penetration.

(3) *Upgrading BI-RADS in malignant masses*

**Fig. 5:**
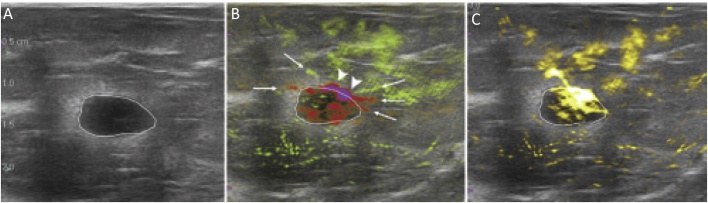
From the Maestro study using the Seno Imagio linear system: a grade II invasive ductal carcinoma that was upgraded from PA/US findings. (A) Photoacoustic US shows findings similar to those from conventional US leading to BI-RADS 4A classification. (B) PA/US shows multiple internal vasculature including deoxygenated (red) vessels with intense deoxygenated anterior boundary zone blush (arrowheads) and boundary zone neovessels (arrows). (C) PA/US total hemoglobin map shows markedly increased hemoglobin within the central tumor. The mass was upgraded from BI-RADS 4A from gray-scale US and US Doppler, to BI-RADS 4C from PA/US. (Reproduced with permission of the authors and publisher.)

PA/US resulted in 1 of 67 malignant masses upgraded from BI-RADS 4A to 4B, a significant 30 of 67 (45%) upgraded from BI-RADS 4B to 4C, and 2 of 67 upgraded from BI-RADS 4B to 5. Fig. 5 shows an example of a BI-RADS 4A from US and US-Doppler upgraded to BI-RADS 4C from PA/US findings.

### 3.2 Technical University of Munich (TUM) hand-held curvilinear system

#### 3.2.1 Instrumentation

The Ntziachristos group developed [66] and recently used a half-arc curved array system called the hand-held MSOT,^1^ to image breast cancer patients [36]. The system appears similar to the one used by the Münster group (Section 3.3) but has notable differences. The TUM hand-held MSOT is more sophisticated in wavelength scanning and uses a higher frequency ultrasound detector with higher aperture. It however lacks the capability to perform ultrasound imaging.

*The ultrasound transducer*. The curved array comprises 256 piezoelectric elements with center frequency of 5 MHz arranged to span 174° with a radius-of-curvature of 60 mm. Parallel data acquisition is performed with a 256-channel A-D converter with 12-bit resolution at sampling rate of 40 MS/s. Real-time image visualization at 50 fps is possible, with the use of the delay-and-sum beamforming image reconstruction algorithm implemented on a graphics-processing unit (GPU).

*The laser and light delivery system*. Illumination on the tissue surface is provided as a sheet (40 × 1 mm^2^) using custom-made fiber bundle (CeramOptec Germany). A frequency-doubled Nd:YAG laser pumping an OPO (Spitlight 600 DPSS, Innolas Laser, Germany) provides <10 ns pulses at a repetition rate of 50 Hz with pulse-pulse wavelength tuning in the range 680-980 nm [66].

*The patient–user interface and measurement protocol*. The transducer is sealed with a membrane, and the space between the active half-arc array filled with heavy water (D_2_O) for acoustic coupling to the subject. The membrane is acoustically and optically transparent.

The breasts of subjects were imaged using clinical ultrasound (Logiq E9, GE Healthcare, Solingen, Germany) with subjects in supine position. Hand-held MSOT was subsequently applied in the same tissue zone, where the lesions had been localized using US. Cross-sectional slices were obtained, for 28 wavelengths from 700 to 970 nm in 10-nm steps. Each slice comprising 28 frames took just 0.56 s to acquire; data for each frame was acquired from a single pulse without averaging. The examination of the region-of-interest took 2-4 min.

*Signal processing and analysis*. In addition to real-time visualization, off-line reconstruction is possible as the raw data is also stored. A model-based acoustic inversion algorithm [67] was applied for the data acquired at different wavelengths. The images were then linearly un-mixed, using the known spectra of the four chromophores: oxy-hemoglobin (HbO_2_), de-oxy hemoglobin (Hb), lipids and water (H_2_O). Four images were thus obtained each corresponding to one of the chromophores. Total blood volume (TBV) was calculated as the sum of Hb and HbO_2_ components. TBV ratios were calculated for ROIs at tumor rim to background tissues, and spatial TBV gradients along profiles were estimated through tumors and healthy tissue.

#### 3.2.2 Patient studies

The predominant goal was to identify the image patterns of malignancies, and investigate image features and (relative) chromophore concentration differences between malignancies and normal breast tissue. Ten patients diagnosed with malignant, non-specific breast cancer (*n* = 8) or invasive lobular carcinoma (*n* = 2) were included in the study. Diagnosis had been made from integrated information from X-ray mammography, US and/or MRI, and core needle biopsy. Tumors were classified by an expert radiologist on the BI-RADS scale, and according to receptor status from immunohistochemistry (HER2, ER and PR).

The following are the most important findings of the small study^2^ :

(1) *MSOT patterns of healthy breast*

**Fig. 6:**
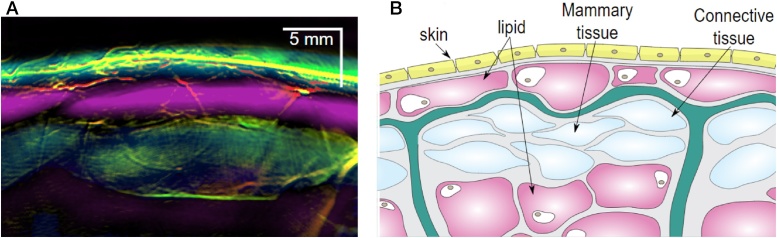
From the Technical University of Munich (TUM) hand-held curvilinear system: (A) composite multispectral photoacoustic (MSOT) image of Hb, HbO_2_, lipid and water, in the healthy breast revealing a layered structure. Colors used: red – HbO_2_; green – Hb; magenta – lipid; blue – H_2_O. (B) Schematic of the organization in the breast: yellow – skin; pink – lipid; light blue – mammary tissue; green - Cooper's ligament. Scale bar 5 mm. (Reproduced with permission of the authors and publisher.)

The sophisticated approaches used, allowed depiction of vasculature and relative chromophore concentrations, such as in Fig. 6(A) where a composite image of the healthy breast shows an overlap of the 4 components – Hb, HbO_2_, lipids and water. This image shows a remarkable agreement with the stratified anatomy of the breast (Fig. 6(B)).

(2) *Enhanced and heterogeneous distribution of vascular signals from cancer*

Tumors show high TBVs distributed heterogeneously, with strong peripheral values and low core values. TBV ratios in all cases of malignancies showed an increase indicating higher vascularization. Intra-tumor variability is shown by TBV spatial gradients in profiles from tumor center to periphery.

(3) *Disruption in MSOT patterns in cancer*

**Fig. 7:**
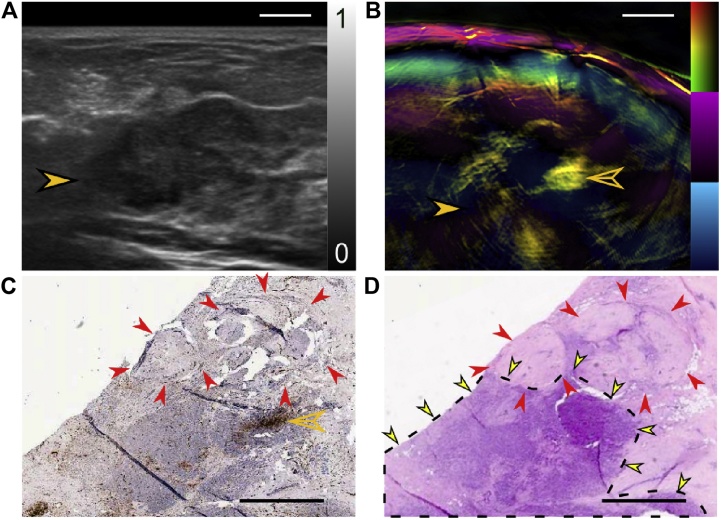
Using the Technical University of Munich (TUM) hand-held curvilinear system: a case of breast with 15 mm × 20 mm invasive lobular breast carcinoma. (A) US image revealing tumor (solid orange arrowhead) centered at 17 mm deep. (B) Composite MSOT image, revealing disruption of the layered organization of tissue around the tumor. Significant is that part of the tumor has low MSOT values, while strong Hb signals are seen clustered in a smaller part (hollow orange arrowhead). (C) Anti-CD31 IHC stained tumor slice in post-surgical pathology confirms high vascularity (hollow orange arrowhead), next to an avascular region (red arrowheads). (D) H&E stained slice adjacent to the one in panel (C) shows carcinoma (dotted line and yellow arrowheads) next to avascular fibroadenoma (red arrowheads). Scale bars (A and B) 5 mm. Scale bars (C and D) 2 mm. (Reproduced with permission of the authors and publisher.)

In addition to the appearance of irregular vascularity, the layered organization, as in (1), can appear disrupted – subtly in some cases and more pronounced in others (compare Figs. 6 and 7 ).

(4) *MSOT and US images carry complementary information*

US and PA images provide complementary information; US imaging showing in cases the entire lesion, while photoacoustics resolves the spatial distribution of vascularity which is more likely associated with malignant processes (Fig. 7).

The authors discuss that MSOT may have potential in identifying infiltration of the tumor into skin, from the disruption in melanin layer and skin vascularity ((1) above). The method may have potential in estimating true cancer mass extent ((4) above), and thereby in biopsy guidance. The high resolutions possible can provide the means to understand spatial tumor heterogeneity and provide valuable fundamental knowledge regarding breast cancer progression and response to therapy.

### 3.3 University Hospital Münster (UKM) hand-held curvilinear system

This group used the MSOT Acuity Echo (iThera Medical, Munich, Germany), a hand-held curved array system with PA and US imaging capability. The device is commercially available from the company for exploratory clinical research.

#### 3.3.1 Instrumentation

*The ultrasound detector*. The hand-held probe has 256 transducer elements for both PA detection and US echography. The elements have a center frequency 3 MHz and a send-receive bandwidth of 56%. The aperture is an arc with an angular span of 125°. A FOV of 40 × 40 mm^2^ in 2-D cross-sectional PA images are obtained with a PA resolution of 250 μm [39].

US pulse-echo imaging is performed by using a synthetic transmit aperture approach. Here within a sub-aperture of 64 elements, 1 element is activated at a time to produce an unfocused wave, with all elements receiving echo-signals simultaneously; beam-focusing is performed in receive. This is repeated for all 4 sub-apertures to acquire the full aperture. The multiple low-resolution images formed are compounded to form the final high-resolution images. The US images have a resolution of 345 μm in a FOV of 40 × 40 mm^2^.

*The laser and light delivery system*. An OPO pumped by an Nd:YAG laser provides 9 ns pulses at a repetition rate of 25 Hz, with wavelengths tunable between 680 and 980 nm. The peak pulse energy is 30 mJ at 730 nm. The laser output is coupled to an optical fiber bundle which terminates via a diffuser in the MSOT probe head. An elliptical shape of beam of 15 × 10 mm is obtained on the target; radiant exposure on the skin is maintained under maximal permissible exposure (MPE) by adjusting pulse energy [39].

*The patient–user interface and measurement protocol*. Both breasts of healthy volunteers (*n* = 6) and breast cancer patients (*n* = 7) were imaged using the hand-held probe. The patients carried histologically-confirmed malignancies and were slated for surgery. Though not mentioned, it is assumed that subjects were in supine position as in conventional breast imaging [39]. Subjects are provided with laser-safety goggles. Examination times were not greater than 15 minutes. Five wavelengths (700, 730, 760, 800 and 850 nm) were applied for the imaging.

US images were used to localize IDC lesions (*n* = 5), and provide ROIs for PA analysis. In the case of DCIS (*n* = 2) due to the absence of specific US features, ROIs were chosen with respect to roughly similarly located ROIs in healthy subjects. In retrospect, it was revealed by MRI that in both cases the DCIS was located in the whole breast.

*Signal processing and analysis*. The MSOT images were reconstructed using filtered backprojection. A first order light attenuation correction in depth was applied assuming an exponential decay with assumptions of *μ*_*s*_ and *μ*_*a*_. A running average of 7 sequential frames was applied to improve SNRs if no detector motion was detected in the sequence. Using spectral unmixing, individual contributions from Hb and HbO_2_ were estimated, and a measures of HbT and oxygen saturation (SO_2_) estimated within ROIs co-localized with US images as mentioned earlier. These MSOT measures were color-coded and overlaid on the US images. Results were compared with findings from MRI images and H&E stained histological analysis.

#### 3.3.2 Patient studies

The goals of the study were to demonstrate clinical applicability of hybrid PA and US imaging, and ascertain the PA presentations of malignancies and healthy breast tissue. Six healthy volunteers, and 7 patients (2 DCIS and 5 IBC cases) were included. The following are the most important findings of the small study:

(1) *Hybrid imaging with US and PA enables co-localization of lesions*

**Fig. 8:**
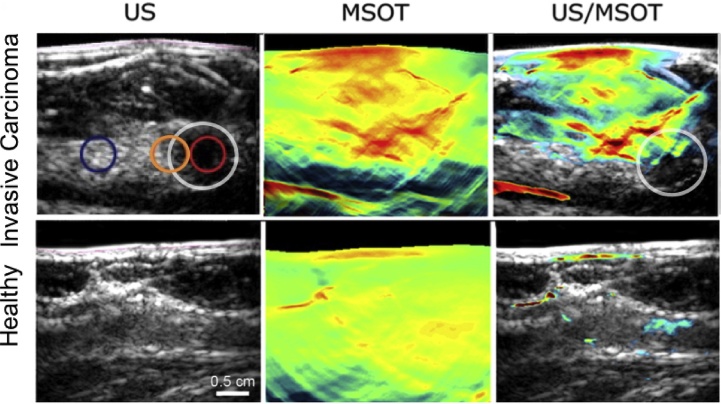
From the University Hospital Münster (UKM) hand-held curvilinear system: US, PA (MSOT) and overlaid US-PA images of a case with invasive carcinoma (first row) compared with a healthy case. The tumour margin, peritumoral tissue and tumor are indicated with respectively orange, red and white circles. Elevated signals are seen in the PA image where tumor is expected. (Reproduced with permission of the authors and publisher.)

Simultaneous recording of reflection US and PA was possible with reconstructions on the fly resulting in a refresh rate of 25 fps at a single wavelength. Frame rates with multiwavelength imaging were possible at up to 5 Hz (25 Hz × 5 *λ*s per image). There appeared to be no relevant operator dependency noted for the handheld imaging of breast carcinoma. All this taken together allowed for an exact anatomical co-localisation and establishing of suspect ROIs using US, enabling in-depth PA analysis of these ROIs (Fig. 8).

(2) *PA signals and estimated parameters reproducible in vivo*

PA signals were detected at depths of 0.5–1.5 cm, and image intensities changed with increasing tissue depth and with wavelength. Estimations of Hb, HbO_2_, HbT and SO_2_ were stable and reproducible in healthy tissue. This suggests that values from healthy subjects may serve as a baseline for assessment of disease as well as monitoring of therapeutic approaches.

(3) *PA derived parameters suggest higher tumor perfusion compared with healthy tissue*

**Fig. 9:**
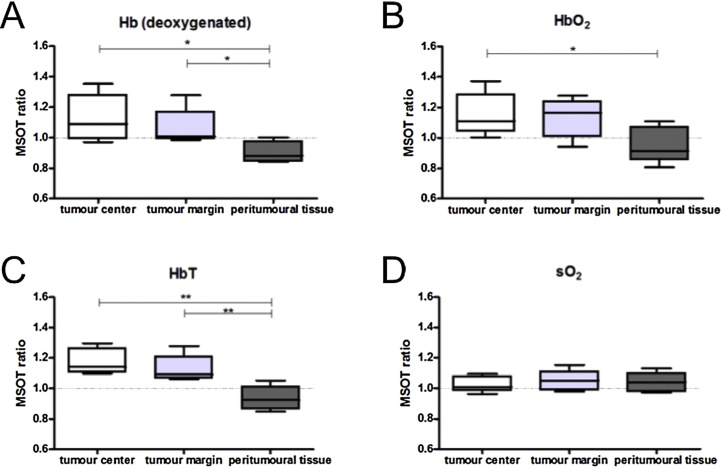
Using the University Hospital Münster (UKM) hand-held curvilinear system: parameters extracted from multiwavelength PA imaging of invasive carcinoma. In invasive carcinoma (*n* = 5) significantly increased tumour-control ratios for (A, B, C) Hb, HbO_2_ and HbT were found in the tumour centre compared with peritumoural tissue. Likewise was found in the comparison of calculated ratios of Hb and HbT in the tumour margin and peritumoral tissue (A, C). No significant differences could be found for oxygen saturation (D). (Reproduced with permission of the authors and publisher.)

Estimated Hb and HbO_2_ values at tumor center and tumor margins showed higher values compared with peri-tumoral tissue. HbT ratios were thus increased at both the tumour centre and tumour margin. The SO_2_ ratios revealed no such significant differences on the other hand (Fig. 9). Albeit from a small number of patients, these results suggest higher tumor perfusion compared with healthy tissue due to tumor neoangiogenesis and inflammation in surrounding tissue. The 2 cases of DCIS did not show any significant increase in any of the MSOT derived parameters.

**Fig. 10:**
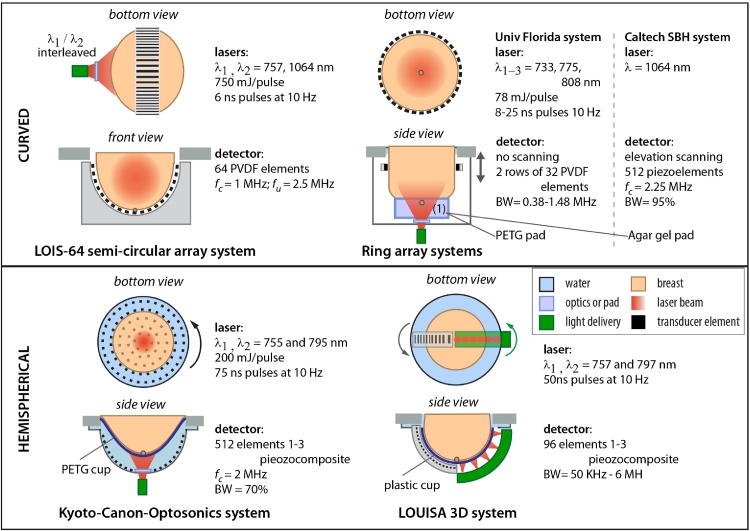
Schematic drawings of Photoacoustic breast imaging instruments with curved, ring and hemispherical detection apertures, with selection of details of their lasers and ultrasound detectors.

### 3.4 The Twente PAM planar system

#### 3.4.1 Instrumentation

This system called the Twente Photoacoustic Mammoscope (PAM), is based on forward-mode or ‘transmission’-mode, where planar US detection is performed antipodal to the illumination side with respect to the breast. This instrument was first presented by Manohar et al. as far back as 2005 [45], with the first patient results reported in 2007 [68]. Yet we describe it as the prime example of the planar approach, with the first peer-reviewed study in human patients. We describe the instrumentation briefly; details can be found elsewhere [45], [69].

*The ultrasound detector*. The planar detector array comprises 590 elements arranged in a roughly circular shape of diameter around 85 mm. The piezoelectric material is PVDF film of 110 μm, the active elements defined by 2 × 2 mm copper pads spring-loading the film against a polymer layer (18 mm thick) which forms the face of the unit. The PVDF elements possess a central frequency of 1 MHz with a fractional frequency bandwidth (FFW) of 130%.

In early work [45], [68], [69], [70], only one element could be selected at a time. In the upgraded version [71], [72], [73], 10 elements could be simultaneously selected. Signals are buffered and pre-amplified using a 10-channel interface unit based on the AD797ar ultra-low noise op amp and the 10 outputs acquired using two 8-channel digitizers (National Instruments, NI PXI 5105, 60 MS/s, 12-bit). The end of cable minimum detectable pressure (MDP) of an element was ascertained to be 80 Pa [45], [74]. With averages over 10 light pulses, an imaging time of 10 min is required for covering the entire detector area of 85 × 90 mm^2^.

*The laser and light delivery system*. In the upgraded PAM system, a Q-switched Nd:YAG laser (Continuum Surelight, California, USA) with pulsed light (10 ns) at a repetition rate of 10 Hz at 1064 nm is used for excitation. The beam was maintained at a fixed position on the breast surface, with a beam area of approximately 35 cm^2^ and an energy of 350 mJ per pulse, giving a radiant exposure of 10 mJ cm^−2^, well below the MPE of 100 mJ cm^−2^ on the skin for the parameters of the light used.

*The patient–user interface and measurement protocol*. A hospital bed was modified to accommodate the instrument. The patient lies prone on the bed with her breast through the aperture [69]. The breast is immobilized under mild compression in a cranio-caudal (CC) direction between a glass window for laser illumination and the US detector array. The detector is mounted on a linear stage that can be manually moved to compress the breast mildly against the glass window. The laser is mounted on the frame of the bed below. To protect bystanders from scattered and/or reflected laser light, a laser safety curtain encloses the instrument and is kept closed during measurements.

The relative position of the compressed breast with respect to the detector array was recorded by making a photograph through the glass window in CC direction. This information was used to overlay PA images on X-ray and MR images (see further). More details can be found in Refs. [71], [72], [73].

*Signal processing and analysis*. The strong breast surface signals are removed from the RF signals by zero padding, so that signals from the PA volume are accommodated within the available dynamic range. Signals were filtered with a band-pass Butterworth filter (cut-off frequencies 0.2–0.7 MHz) [69]. Reconstruction was performed using an acoustic backprojection algorithm, using a homogenous speed of sound (SOS) of 1540 m s^−1^ in the breast. The resolution of the imager is roughly 3.5 mm in both axial and lateral directions at a depth of 30 mm assessed from earlier phantom measurements.

Sagittal slices of the reconstructed volume were Hilbert transformed, and voxel intensities normalized to the maximum intensity in the volume. The relative position of the breast and detector array from the photograph allow the location of the PA FOV in the breast to be ascertained. The orientation and size of the breast from the photograph are adjusted to match the CC X-ray image and the MR image [71], [72], [73]. This permits the MIP of the reconstructed PA volume to be overlaid on the X-ray and MR images. When a co-location of the PA intensity distribution with the lesion in X-ray was found, the PA feature was judged to be originating from the abnormality, and is referred to as a PA lesion. The lesions were classified according to their appearance on the MIP. The size of the PA lesion is estimated via a manually drawn contour around the lesion on the MIP using information from conventional imaging.

#### 3.4.2 Patient studies

In a proof-of-principle study in 2007, Manohar et al. [68] observed higher intensities in PA images in 4 of the 5 malignancies studied. Since then in an expanded study a further 51 abnormalities have been imaged. These lesions cover 41 carcinomas, 7 cysts, 2 fibroadenomas and 1 chronic active inflammation.

The following are the most important findings:

(1) *PA breast imaging shows high contrast for infiltrating ductal carcinoma (IDC)*

From the study in 2012 [70], on highly suspicious (BI-RADS 5) breast lesions, it was concluded that in all 10 IDC studied, the lesions could be visualized with high contrast (average 5.0) in confined regions. From the 2015 study reported in Refs. [71], [72], 30 of 31 malignancies (predominantly IDC) could be identified, with an average contrast of 3.6. In all cases the PA images were compared with X-ray images, US images and conventional histopathology.

(2) *Breast malignancies show signature PA presentations*

**Fig. 11:**
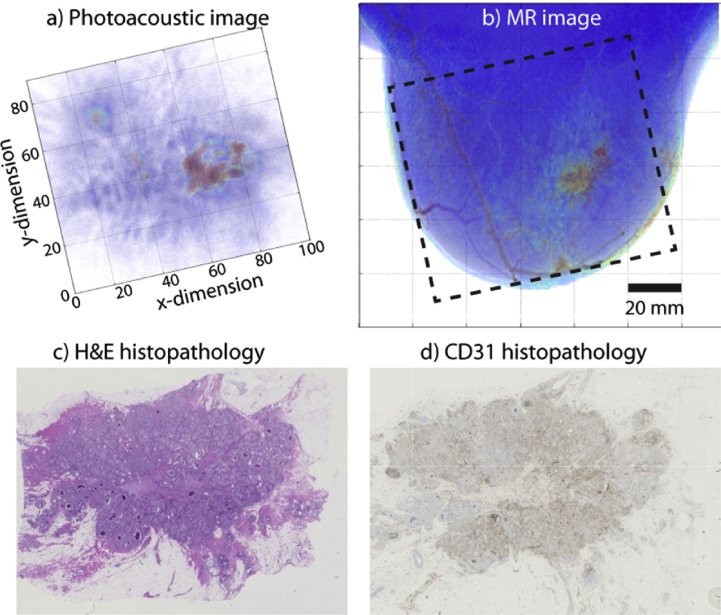
Using the University of Twente PAM planar system: example of PA mass appearance seen in 63 year old patient with infiltrating ductal carcinoma (IDC). (A) The breast was tilted during the PA measurement to position the lesion favorably within the detector's FOV of 85 × 90 mm^2^. The lesion in the average intensity projection (AIP) PA image, is visible as an irregular, high contrast, 29 mm mass. The lesion co-localized well with the lesion in the X-ray image (not shown). The lesion also co-localized well with (B) the AIP MRI image after tilting the PA image. The dashed box indicates the FOV where PA image is acquired. The MR appearance is described as an irregularly shaped mass (C) post-surgery H&E stained specimen revealed the presence of a 34 mm grade 2 IDC. (D) The CD31 stained tumor slide shows the microvascularity spread over the entire lesion supporting the mass-appearance observed in PA and MR images. It is intriguing that the patterns in (A)–(D) appear roughly similar in appearance. (Reproduced with permission of the authors and publisher.)

PA appearances of IDC, and their correlation with tumor vasculature were investigated in Ref. [70]. PA images were compared with MR images and with vascular staining in histopathology, in addition to X-ray and US imaging. Similar PA appearances as contrast enhancement types reported in MRI of breast malignancies were observed, albeit in the small cohort, namely: (i) mass appearance; (ii) ring appearance; and (iii) non-mass appearance. MR images were available for a total of 11 cases, and correspondence was very good to excellent.(Fig. 11) In 6 cases, CD-31 immunohistochemistry (IHC) in histopathology, showed good correspondence between density and distribution of vascularity, and PA image patterns (Fig. 11).

(3) *Breast cysts show specific PA appearances*

Using 1064 nm excitation, cysts were visible as either one or two confined high contrast areas representing the front, and the front-and-back of the cyst respectively [75] (not shown in this review). The PA appearance depended on the absorption contrast between cyst contents and embedding tissue. These features were found to be due to an abrupt change in absorbed energy density and Grüneisen coefficients across the interfaces, in combination with the limited-view geometry. Cysts can thus be mistaken for malignancies which can also appear as one or multiple confined contrast areas.

### 3.5 The Kyoto-Canon planar geometry system

#### 3.5.1 Instrumentation

The Kyoto-Canon group reported on the PAM-01 planar PA breast imager in 2011 [76]. The system can simultaneously or separately illuminate the breast from a forward and backward direction toward an array detector. In succession, the PAM-02 system was developed, which uses in addition to the US detector array, a dedicated US linear probe for echo imaging [77]. By this, non-invasive imaging of morphology (from US) and function (from PA) becomes possible.

*The ultrasound detector*. PAM-01 uses a custom-made piezocomposite transducer (Vermon S.A., France) comprising 345 elements (15 × 23) with sizes of 1.8 × 1.8 mm^2^. These elements are arranged with a pitch of 2 × 2 mm^2^ in a rectangular grid to provide an aperture of 30 × 46 mm^2^. To cover a larger zone of the breast, the array is mechanically scanned. A center frequency of 1 MHz (80% BW) was chosen as a trade-off between ultrasound attenuation and image resolution [48]. A custom-made 345-channel DAQ was used to acquire the signals.

PAM-02 uses a different detection array, based on highly promising CMUT technology. Here 600 elements (20 × 30) with sizes of 0.8 × 0.8 mm^2^ and pitch 1 × 1 mm^2^ elements are arranged in a rectangular grid of 20 × 30 mm^2^. The elements have a center frequency of 2 MHz with a wide bandwidth of 130% FFW. The noise equivalent pressure (NEP) was measured to be 5.6 Pa. This new detector helped in improving the spatial resolution from 2 mm in PAM-01 to 1 mm in PAM-02.

A dedicated 128 element linear transducer array was added for pulse echo imaging, making this the first system with hybrid PA and US imaging. The transducer array for US imaging has a 6 MHz center frequency and an 80% FFW [77].

*The laser and light delivery system*. Both systems illuminate the breast from two sides to achieve higher fluence deep inside the breast. A tunable Ti:Sapphire laser optically pumped by a Q-switched Nd:YAG laser is used, with 7 ns pulses at a frequency of 10 Hz. Patient measurements were performed at 756, 797, 825 and 1064 nm using PAM-01 [48]. From these experiences, the authors concluded that the optimum wavelengths for PA visibility and Hb saturation calculations are 797 and 756 nm. Therefore, only these two wavelengths were utilized in the PAM-02 system [77]. The ‘backward’ (same side as detector array) laser generated 60 mJ/pulse and illuminated the breast, without overlapping, via two pathways, from the left and the right side of the PA transducer. The ‘forward’ laser uses 85 mJ/pulse. These energies were constant for both wavelengths. Radiant exposures of 15 and 11 mJ cm^−2^ were set for the forward and backward laser respectively [77].

*The patient–user interface and measurement protocol*. In PAM-01, the patient lies on an examination table in prone position with her breast pendant through an aperture. Under the table, the breast is mildly compressed in a cranio-caudal direction between two transparent 10 mm thick plates. Acoustic coupling gel is applied between the breast and the plates, and the detector array contacts the caudal plate [48]. The caudal plate is composed of polymethylpenthene (PMP) to reduce the acoustic attenuation. The other plate is composed of polymethyl methacrylate (PMMA), which has a high light transmittance. In the PAM-01 set-up, scans with a measurement area of 30 × 46 mm^2^ are made in 45 s. For first measurements with the system, scans were made of three adjacent areas in the tumor location. Later studies used automatically translation to provide a coverage of 120 × 46 mm^2^ area.

In PAM-02, the patient user interface was revised. The bed was widened, such that patients could lie in different angles with respect to the aperture to make scans in both the cranio-caudal as the medio-lateral oblique direction. Secondly, the breast holding plates were reduced in thickness to reduce attenuation and a nanocomposite gel was used as coupling between the breast and the PMP plate. The gel remained attached to the plate, which is the main advantage over the conventional US coupling gel, and adapts easily to the shape of the breast. Finally, the maximum scan area was increased to 150 × 90 mm^2^. The linear array, used for US echo imaging, is placed next to the PA detector, and is translated to image a wide area of the breast [77].

*Signal processing and analysis*. A modified 3-D universal backprojection algorithm [78] was used for reconstruction, with corrections for sound speed and refraction through the PMP plate. Under the assumption of homogeneous background absorption (*μ*_*a*,*b*_) and reduced scattering ($\mu_{s,b}^{\prime }$), the fluence distribution at the PA pressure locations was approximated by the diffusion approximation. It appears [77] that values of *μ*_*a*,*b*_ and $\mu_{s,b}^{\prime }$ per breast were estimated using time-resolved spectroscopy, applied in breast volumes far from lesion locations. From this optical absorption factor at the PA sources, ${\tilde{\mu }}_{a}$ calculated in this way for the two wavelengths. From this a blood oxygen saturation index (S-factor) was calculated, knowing the molar extinction coefficients of Hb and HbO_2_ from the literature. An S-factor image was developed using intensity assigned to the ${\tilde{\mu }}_{a}$ at 795 nm as being proportional to total HB concentration, and with a color hue assigned to the S-factor value.

A 3-D US image in PAM-02, was constructed by combining individual B-mode images acquired by the scanning probe. The resolution was improved by synthetic aperture imaging, and further by making use of the Capon method [79]. The resolution of the US images was <1 mm. In the end, a fusion image was made by overlapping the PA and US images [77].

#### 3.5.2 Patient studies

PAM-01 was used for a first clinical study to study the utility of the prototype in imaging breast cancer and in extracting functional information in tumors in comparison with conventional imaging and histological assessment of angiogenesis and hypoxia. Results from a total of 39 lesions malignancies were reported [48], [80].

Details of the hybrid version PAM-02, were reported by Asao et al. [77], in which one clinical case was discussed in great detail, mainly to illustrate the capabilities of the new imager.

Here we summarize the most important findings of the studies:

(1) *Tumor-related vasculature but no ‘masses’ visualized*

In a case discussed in Kitai et al. [48] using PAM-01, sparse clusters of higher intensities are observed at the tumor periphery. The locations match with rim-enhancement features in contrast-MRI. In a second case, similar PA presentation is observed, while MRI shows mass-enhancement.

Using the improved PAM-02 in the 1 case discussed [77], the PA images and the images combining S-factor with the PA data, shows sparse spotty signals within the tumor. However, the most dominant structures appear to be blood vessels, centripetally approaching the tumor, and getting disrupted at the borders [77].

A discussion point in Ref. [77] is that PA signals inside tumors tended to be lower inside the tumor than outside at the periphery, despite absence of tumor necrosis. Further research from both biological and technical viewpoints is required to understand this.

The visibility of malignancies based on visual assessment of breast and tumor vasculature as above, reported in the PAM-01 study [80] is approximately 75%. This implies that not all tumors showed the specific patterns relating to a micro-vessel rich lesion. The reason for this could be biological or technical, and needs to be further researched.

(2) *Hybrid PA and US imaging provides superior lesion identification*

**Fig. 12:**
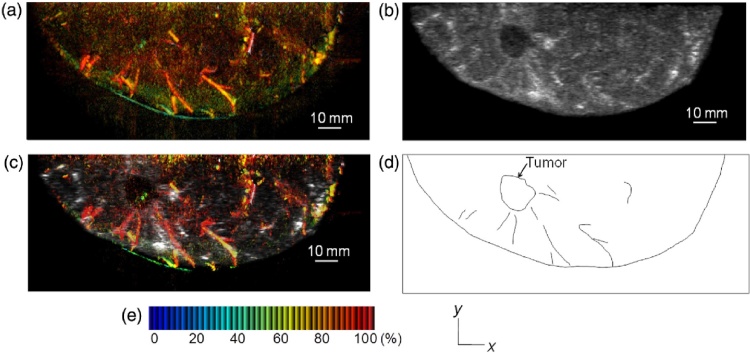
Using the Kyoto-Canon planar geometry system PAM-02: (A) S-factor distribution image, (B) US C-mode image, and (C) S-factor distribution image overlaid on the US C-mode image. The color image represents the S-factor distribution image, and the gray-scale image represents the US C-mode image. (D) Schematic illustration of the tumor location. (E) Color scale for S-factor value. The color changes from blue to red as S-factor increases from 0% to 100%. (Reproduced with permission of the authors and publisher.)

Ref. [77] demonstrates the importance of using simultaneous US imaging to identify lesions. The US data allows to pinpoint tumor zones where the PA images carry information relevant to tumor-related vascularization. The PA signals can be analyzed to determine angio-architecture inside and outside the tumor and estimate vessel densities and blood oxygen-saturation (Fig. 12).

(3) *Oxygen saturation in malignancies lower than in subcutaneous vessels*

The S-factor value weighted with the local *μ*_*a*_ value is a measure of blood oxygenation saturation (Fig. 12). The first results indicate that this value is lower in malignancies (28 lesions) compared to that in the subcutaneous vessels. On the other hand, the estimated S-factor value in subcutaneous vasculature in both tumor-bearing breasts and contralateral breasts is comparable. The value is also lower (22 lesions) compared with corresponding ROIs in the contralateral breast. Taken together, this suggests that malignancies have reduced tissue oxygenation and hypoxia compared with normal tissue.

### 3.6 LOIS-64: semi-circular aperture imager

#### 3.6.1 Instrumentation

The curved geometry for breast imaging was introduced by the Oraevsky group [34], [81], [82]. Here we describe the Laser Optoacoustic Imaging System (LOIS-64) using a 64-element arc array of rectangular transducer elements [49]. In spite of the fact that this instrument was developed in 2009, we describe it as a prime example of the semi-circular aperture, especially since various strategies and approaches used for the challenges faced are still relevant today.

*The ultrasound transducer*. The detection array is an arc with a 180° aperture and radius of curvature around 45 mm. It comprises 64 rectangular detectors of polyvinylidene fluoride (PVDF) with sizes 20 × 3 ×0.11 mm. The frequency bandwidth is extremely broad, the −6 dB response to an impulse extending from a few hundred kHz to 2.5 MHz [49]. The signals are amplified by a low-noise two-stage amplifier, designed in house, to respond to this wide bandwidth of the PA signals. An individual channel has an average sensitivity of 1.66 ± 0.21 mV/Pa at 1.5 MHz. The arc-array spans the breast (resting in the array) either in a medio-lateral or cranio-caudal axis depending on its orientation. The illumination is orthogonal to the array allowing 2-D slice images to be made with an in-plane resolution of 0.5 mm and a thickness of 20 mm.

*The laser and light delivery system*. The light excitation is with a Q-switched Alexandrite laser delivering 75-ns pulses (750 mJ/pulse) at 757 nm with a repetition rate of 10 Hz. The light is coupled to the breast through a fiber bundle and beam-expander, to provide 70 mm diameter at the surface with a radiant exposure of 10 mJ cm^−2^.

*The patient–user interface and measurement protocol*. The detector array represents a hemi-cylindrical cup in which the breast is suspended through the aperture in an examination table on which the patient lies prone. The cup had a radius of its cylindrical surface of 70 mm and width of 90 mm.

After ascertaining safety in a first phase, 27 patients were included to study the capability of LOIS to visualize breast cancer. Patients with a suspicious lesion identified in X-ray mammography and/or US imaging, and scheduled for breast biopsy, were included. Prior to the biopsy, imaging with LOIS was performed. A tumor was deemed to be visible in the PA image, if an isolated area of increased intensity could be localized in the quadrant of the breast suspected to contain a tumor according to the conventional imaging results. The final diagnosis was made based on the biopsy results.

*Signal processing and analysis*. The characteristic *N*-shaped temporal form of the PA signal suggested to the authors the use of the wavelet family in general, to extract the signals from noise, interference and artifacts. The third derivative of the Gaussian wavelet was found to be the best candidate for filtering the signals, providing a monopolar pulses while significantly reducing artifacts (see further).

The filtered pressure signals were weighted for the directivity of the individual detectors and used to reconstruction images of using the radial back-projection algorithm [83]. Image visualization could be performed in real-time at 1 fps for 512 × 512 pixel images and 10 fps for 128 × 128 pixel images.

#### 3.6.2 Patient studies

The PA images correctly identified 17 from 25 carcinomas during the study. In 6 of the 8 cases where the tumors were not detected, technical malfunction and operator error had taken place. In 2 cases the reconstructed lesions showed insufficient contrast to be unambiguously identified.

The following are some of the highlights of the work:

(1) *LOIS was capable of visualizing breast cancer with high contrast*

This work clearly demonstrated the feasibility of using PA imaging as a high-contrast modality for imaging of breast cancer. Considering all technically acceptable acquisitions, LOIS visualized 18 of 20 malignancies confirmed by biopsy.

(2) *The performance of LOIS was superior to X-ray mammography in several cases*

**Fig. 13:**
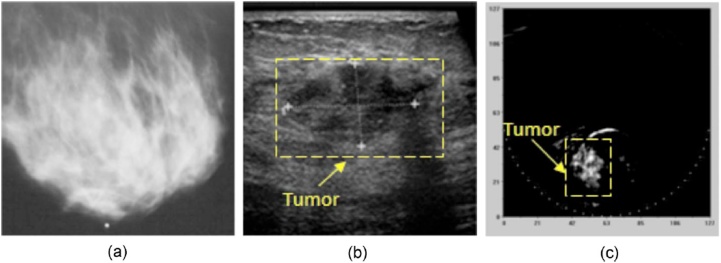
Using the LOIS-64 – semi-circular aperture imager: a case of breast with an infiltrating ductal carcinoma. (A) The lesion is occult in the radiographically dense breast in the X-ray image. (B) The US image reveals the tumor at 21 mm deep. (C) The LOIS image reveals the lesion with strong contrast, pointing to vascularization which is indicative of a malignant tumor. (Reproduced with permission of the authors and publisher.)

Of the 18 LOIS visualized carcinomas, 5 were occult in mammography, while only 1 tumor seen in mammography was not visualized. In Fig. 13, is the case of a poorly differentiated IDC grade 3/3. The breast is radiodense and the tumor cannot be identified in X-ray mammography. In US imaging the tumor is visible as a 23 × 15 mm lesion at a depth of 21 mm. The tumor was unambiguously identified in the PA image at the correct location with a tumor-background contrast superior to that in US imaging.

(3) *Possibility and necessity for hybrid PA and US imagers*

The authors discussed the benefits of synergy between PA and US imaging. Echography is never used in isolation in breast imaging due to high false-positive rates and operator dependence. PA imaging can be combined with US imaging since the acoustic hardware required for both techniques can be shared. The combination of the two complementary techniques, would especially have impact in case of the dense breast. In the hybrid system, US would provide information about acoustic boundaries, while PA imaging would map the vascular distribution in the lesion.

(4) *Improvement in signal detection with the use of wavelet-based filtering*

This work also demonstrated the application of wavelet analysis to recover the transient PA signals in the presence of ‘acoustic’ artifacts. These interfering signals are caused by light arriving at the detectors's surface after interaction with the object, and due to the strong fluence at the surface of the object. Both interfering signals are large-magnitude low-frequency signals, that dominate smaller PA pulses from within tissue and from tumors. It was shown that by using different scales of the analyzing wavelet, the transform was able to distinguish between the short duration (higher frequency) of the PA signals of interest, and the long duration (low frequency) artifacts.

### 3.7 University of Florida ring-shaped imager

#### 3.7.1 Instrumentation

The Jiang group developed their system to provide functional information of breast tissue using multi-wavelength illuminations and model-based reconstruction of the tomographic data. This work presents the first images of quantitative hemoglobin concentration and oxygen saturation, in healthy and afflicted breasts [84].

*The ultrasound transducer*. The ring-array, developed in-house, comprised 64 ultrasound detectors based on PVDF film (110 μm) arranged in 2 rows of 32 elements each with size 2.3 × 30 mm. The −6 dB bandwidth of each transducer extended from 380 kHz to 1.48 MHz [50], [85]. The array is coupled via a multiplexer to a 16-channel preamplifier and DAQ system sampling at 50 MS/s.

*The laser and light delivery system*. Light was delivered to the breast from the nipple side through a transparent PETG plate from under an examination table on which the subject lies prone (see further). The light source is a tunable pulsed Ti:Sapphire laser pumped with a Q-switched Nd:YAG laser, and provides 8-25 ns pulses at a repetition rate of 10 Hz. The beam is expanded to 6 cm^2^ and made diffuse with ground glass to achieve a radiant exposure of 13 mJ cm^−2^ at the breast surface. To achieve whole breast imaging, the beam is scanned mechanically on the breast surface [84], [50], [85].

*The patient–user interface and measurement protocol*. The system is built into an examination table on which the subject lies prone. The ring-array is radially adjustable so that the diameter can be adjusted to fit different breast sizes. It appears that the detectors directly contact the breast; no information is provided about the exact acoustic coupling. A PETG plate at the bottom through which light falls on the breast can be elevated. The breast hangs into aperture, where it is slightly compressed by the PETG plate moving upwards, and by the ring-array with individual detector-pairs moving inwards to achieve a cylindrical form with diameter around 10 cm and an elevation thickness of less than 6 cm.

Ten subjects participated in the study – 4 healthy, 2 with DCIS, and 4 with IDC. The six cancer cases were diagnosed at the time of enrollment, and X-ray, US, and MRI images and pathology results were available for validation. The HbT and SO_2_% maps were evaluated separately without a priori information from any other clinical findings.

*Signal processing and analysis*. A finite element method (FEM) based image reconstruction algorithm with total variation (TV) minimization was used for recovering HbT and SO_2_%. Details and validation can be found in Refs. [50], [85], [86], [87]. The spatial resolution of the system was 0.5 mm within the imaging plane based on phantom experiments. The slice thickness is 30 mm due to the elevational dimension of the ultrasound detectors.

#### 3.7.2 Patient studies

The patient studies show the first quantitative Hb concentration and SO_2_ images in the human female breast. The following are the highlights of the small study:

(1) *PA images were consistent with MR images*

The proof-of-principle study suggests that the PA images provide superior or similar resolution in lesion depiction to MRI, and that that PA is at least as sensitive as MRI.

In all 6 breast cancer cases, tumor regions could be visualized on the basis of HbT and SO_2_ maps. This visualization and localization was grossly similar to MR results. In 5 of these cases, the results in both PA and MR were consistent with pathology findings. The one ‘false positive’ case in PA and MR was imaged shortly after NAC, while surgery and post-surgical pathology was performed much later. Changes in vascularization may have thus not yet kicked in at the time of imaging.

**Fig. 14:**
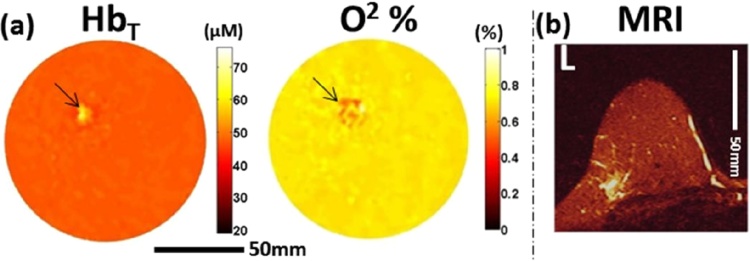
From the University of Florida ring-shaped imager: High grade IDC in the left breast. (A) Coronal HbT and SO_2_ maps from PA imaging through the lesion. (B) 2-D slice of T2-weighted MRI through same region taken 3 days before PA examination. (Reproduced with permission of the authors and publisher.)

Fig. 14 shows an example of the PA imaging results on an IDC. The HbT map in the coronal plane of the lesion located in the breast upper outer left quadrant shows high contrast in a 1.1 × 0.8 cm heterogeneous area. This pattern is also recognized in the SO_2_ map, where the value in the lesion is considerably lower than the average. The dimensions of the contrast regions in both images are consistent with the T2-weighted MR image (1.8 × 1.4 × 2.2 cm).

(2) *PA is capable of sub-millimeter resolutions in maps of tumor functional information*

**Fig. 15:**
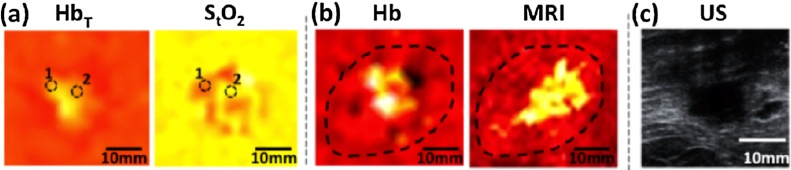
From the University of Florida ring-shaped imager: detailed images of the case in Fig. 14. (A) coronal HbT and SO_2_ maps; (B) Hb map and T2-weighted MR image; and (C) US image. (Reproduced with permission of the authors and publisher.)

The resolution achieved in the system, and the image reconstruction algorithm employed, make possible detailed images perhaps at the level of microvessels. For example the same case as Fig. 14 is detailed in Fig. 15. Here HbT, SO2, and Hb maps are shown zoomed in on a ROI of 55 × 55 mm, and compared with MR and US images. The roughly oval shaped region is visible in various parameters across all techniques, with apparently higher detail in the PA derived images.

(3) *Heterogeneous patterns in PA derived parameter maps*

Related to the image performance in (2) above, the authors show heterogeneous patterns in HbT, SO_2_ and Hb maps. With reference to Fig. 15, the oval-shaped region in the Hb map is irregular with low and high values, correlating with features in the MRI image. Similarly for the SO_2_ images, elevated and low values are seen, as also in other cases discussed by the authors. While more detailed studies need to be done, this may be revealing the intrinsic tumor heterogeneity.

(4) *Possible improvements in detection accuracy and use of more wavelengths*

The system has anisotropic resolutions with an in-plane resolution of 0.5 mm, but a slice thickness of 30 mm. While the large active area of the detectors improves the signal detectability, the appearance and the accuracy of the quantification are strongly perturbed as the signals carry information from both normal and diseased tissue within the imaging volume.

Further, H_2_O and lipids contribute to the signals generated in addition to Hb and HbO_2_, but are not taken into account in developing the reconstructed parameter maps. Authors have the opportunity to measure the actual tissue scattering coefficient distribution, using diffuse-optical tomography in a hybrid system [88], [50], [85], which will improve the quantitative reconstruction further.

### 3.8 Caltech single-breath-hold ring-shaped imager

#### 3.8.1 Instrumentation

The system is capable of volumetric full-breast PA imaging within 15 s – a single breath hold, and real-time 2-D cross-sectional slice imaging. By this PA angiography can be performed with no breathing-induced motion artifacts. In the real-time mode, new capabilities open up such as observation of pulsatile blood-flow allowing identification of arteries from veins, and photoacoustic elastography.

*The ultrasound transducer and light delivery*. The system is equipped with an 512-element unfocused full-ring detector array (Imasonic SAS, Besancon). Each element has a 2.25 MHz center frequency and a >95% bandwidth. Elements are 5 × 0.65 mm^2^ sized with an inter-element spacing of 0.7 mm. The aspect ratio yields anisotropic resolutions in-plane and elevational respectively of 255 μm and 16.1 mm in 2D. The latter is due to the 9° divergence angle in the elevation plane. The array can be maintained at a certain fixed elevation position for 10 Hz imaging. For full-breast imaging, the array can be translated from the chest wall towards the nipple in 15 s. With the 3D data set and the 3D backprojection algorithm, the elevational resolution improves to 5.6 mm, around 3 times finer than that from the 2D data set and 2D reconstruction algorithm

The breast is illuminated from the bottom by 1064 nm pulses in a donut-shaped beam using an Axicon lens and a diffuser to deposit less energy at the pigmented nipple-areolar complex, and to provide a homogeneous fluence deeper in the breast. A penetration depth of 4 cm has been achieved with this strategy.

*The patient–user interface and measurement protocol*. The patients are positioned in prone position on a bed with the breast hanging through an aperture. A compression pillow made of agar, located at the bottom of the imaging tank, slightly compresses the breast against the chest wall. This provides a lower breast thickness for light to penetrate deeper coming in from the bottom. The imaging tank is filled with water at a temperature of 35 °C. The complete imaging study, involving imaging both the ipsilateral and contralateral breasts took less than 10 min. For PA angiography, patients were asked to hold their breath; within 15 s, the detector array is scanned from nipple to the chest wall. For elastography, patients breathe normally causing the breast to be pushed against the agar pillow, elevationally generating a deformation of the breast in the coronal plane. The elastographic measurement is performed in a single cross-section during 10 s.

*Signal processing and analysis*. The data is acquired by a DAQ with 4 sets of 128 channels with pre-amplification. PA signals are recorded for 100 μs after each laser pulse. Both 2D and 3D reconstructions are obtained using the half-time universal back-projection (UBP) algorithm [89]. To enhance contrast of blood vessels, Hessian-based Frangi vesselness filtration [53] was used. Images were color-coded on depth from where signals were measured. Methods were also applied for extracting vascular diameters, and for monitoring pulsatile arterial fluctuations.

A tumor demarcation algorithm was developed to quantify the vessel density distribution inside breast, enabling the visualization of tumors. In the elastography mode the 2-D strain was estimated by evaluating area-quantification grids between vessels, as tissue deformed slightly due to breathing. This allows the extraction of the compliance between tumors and surrounding normal breast tissue.

#### 3.8.2 Patient studies

**Fig. 16:**
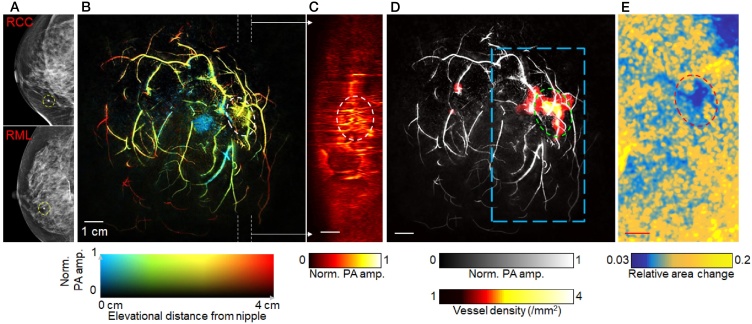
From the Caltech Single-Breath-Hold ring-shaped imager: (A) X-ray mammograms of a 69-year-old female patient with an invasive ductal carcinoma (grade 2/3). RCC, right cranial-caudal; RML, right medio-lateral. (B) Depth-encoded PA image. (C) Maximum amplitude projection (MAP) images of thick slices in sagittal plane. (D) Automatic tumor detection on vessel density maps. (E) Maps of the relative area change during breathing. (Reproduced with permission of the authors and publisher.)

After imaging studies on phantoms and a healthy volunteer, seven breast cancer patients were included. These women had differing skin colors (light to dark), various breast sizes (cup B to DD), various radiological breast densities and various lesion types. The tumors ranged in size from 1 to approximately 3.2 cm. Fig. 16 shows the case of a 69 year old patient with IDC. The PA image in (B) shows the depth-encoded angiogram with the tumor in encircled region, and (C) shows maximum amplitude projection (MAP) images of thick slices in sagittal planes marked by white dashed lines in (B). Automatic tumor detection on vessel density map is shown in (D), with the tumors identified by green circles. Background images in gray scale are the MAP of vessels deeper than the nipple. From the elastography mode, the map of relative area change during breathing (in the regions outlined by blue dashed boxes in the angiographic images in (D)) is shown in (E). The same tumors are identified by red circles. The tumor locations in all the images correlate well with the findings from X-ray imaging (Fig. 16(A)).

The following are the highlights of the study:

(1) *PA image derived angiographic densities reveal tumors*

Eight of nine biopsy-verified tumors were detected based on identifying higher blood vessel densities in the breast. The identified tumor regions showed a vessel density contrast between tumors and normal breast tissue of roughly 3.5. The quantification of tumor vascular densities also aided lesion discrimination, with vessel density ratios of the six malignancies 1.4 times higher than that of the two benign lesions. In the small cohort, with appropriate tuning (manually as well as automatically) of thresholds between high and average vascular densities, a true positive rate as high as 87% and a true negative rate as high as of 86% could be obtained.

(2) *Photoacoustic elastography improves lesion detection*

The one tumor that was not detected using vascular densities was located unfavorably from a laser fluence exposure point-of-view, being in a poorly illuminated marginal region of a D cup breast. This suggests that illumination should be optimized in the Caltech instrument, but also points to the more general challenge of achieving sufficient fluence for full-breast coverage. The important point in this work, is the addition of photoacoustic elastography, which enabled the detection of the otherwise occult tumor. In the complete study, elastography identified all six tumors in the five patients studied. The average difference in compliance in tumors was around 2 times lower than that in normal breast.

(3) *Comparison with conventional imaging*

The in-plane resolution 255 μm is approximately four times finer than that of 1.5 Tesla standard MR imaging. In the small study presented, the Caltech system could delineate a tumor as small as 8 mm; standard MR and X-ray imaging can visualize tumors as small as 4 mm. In general, one can say that the imager characteristics and results suggest that the PA images will be able to provide at least similar resolution in lesion depiction to MRI and X-ray imaging, but this will have to be shown in an expanded study. In the study, photoacoustics could clearly reveal tumors in radiological dense breasts, while these were only barely distinguishable using X-ray imaging.

(4) *Appropriate technical choices crucial for identification of tumors*

The choice of imager specifications in terms of imaging geometry, detector number and DAQ speeds for achieving fast acquisition on the one hand, together with detection characteristics and manner of illumination on the other, enable highly optimized and sensitive *in vivo* measurements to be made. Additionally this work has shown that merely observing contrast is not sufficient to identify tumors, rather algorithms have been developed to enhance vasculature, estimate vessel diameters and extract vessel densities from acquired and reconstructed data. The end result has been excellent depiction of blood vessels, and more importantly the identification of tumors and malignancies. Further, the additional functionality of elastography imaging, made possible by the high spatiotemporal performance and development of appropriate algorithms for estimation of compliance, provides an additional powerful contrast mode in the imager, using the same data acquisition with a slightly modified patient protocol.

### 3.9 LOUISA 3-D hemispherical imager

#### 3.9.1 Instrumentation

This description is based on a short-conference proceeding from the group of Oraevsky [90]. With experience from the LOIS-64 system [49] and the 3-D preclinical imager LOUIS-3-DM [91] a clinical prototype for 3-D breast tomography was developed. The Laser Optoacoustic Ultrasonic Imaging System Assembly (LOUISA-3-D) is capable of full breast view functional imaging enabled by two rapidly toggling laser wavelengths in the NIR spectral range. An ultrasound imaging module also provides that allows a sequence of B-mode ultrasound slices to be made.

*The ultrasound transducer and light delivery*. The imaging module comprise an arc-shaped US detector array module with 96 ultrawide-band detectors embedded in a 16 cm diameter hemispherical imaging bowl. The detectors covering a 90° arc, are based on a piezocomposite material. The detectors have a frequency band extending from 50 kHz up to 6 MHz with a noise equivalent pressure of 1.3 Pa and a sensitivity of 0.012 mV/Pa. The overall spatial resolution for the system is 0.3 mm.

For B-mode US imaging a second 90° arc-shaped array is used with 192 transducers with a 7 MHz center frequency and 100% bandwidth. With this 2-D slices of breast anatomy are acquired, which can be overlaid with matching optoacoustic slices selected from the 3-D images.

The system is equipped with an Alexandrite laser with the ability to interchangeably emit 757 and 797 nm pulses with a pulse width of 50 ns and 10 Hz repetition rate. The laser is connected to an arc-shaped fiber-optic delivery system, which can rotate around the imaging bowl independent of the detector array.

*Patient–instrument interface and protocol*. The patient lies in prone position on the examination table with her breast through an aperture and stabilized in the imaging bowl, which contains an acoustic coupling medium. The wall of the bowl is made of a thin polymer and is optically and acoustically transparent. As mentioned, the detector array is embedded in the wall to have the active elements flush with the inner surface. For a certain position of the array, the light delivery system makes 10 rotational steps to illuminate the entire breast, with detector signals integrated for this projection. Subsequently, the entire imaging module comprising imaging bowl, detector and light delivery array is moved to a new position around the breast. This is repeated for a total of 320 positions. For two wavelengths, the theoretical minimum time for a 10 Hz interleaved scan is around 10 min per breast.

*Signal processing and analysis*. The detector signals are acquired by a multichannel DAQ system with 70 dB analog amplification, connected to a computer for signal control, data processing and image reconstruction in spherical coordinates. With this system, tumors with a typical *μ*_*a*_ of 10 mm^−1^ illuminated with a fluence of 0.01 mJ cm−2 can be recovered. Such a fluence is expected at a depth of 5 cm with a safe fluence of 20 mJ cm^−2^ at the skin. Due to the large amounts of data being collected (320 angular positions × 96 elements × 1536 data samples) image reconstruction is a computationally intensive job, but is performed in about 4 min. After the image reconstruction an inverse attenuation function (*μ*_*eff*_ = 1.15 cm^−1^) is applied to the voxel brightness in radial direction from the skin surface towards the center of the breast to compensate for optical attenuation. For the B-mode ultrasound image, 2-D reconstruction is performed.

#### 3.9.2 Patient studies

**Fig. 17:**
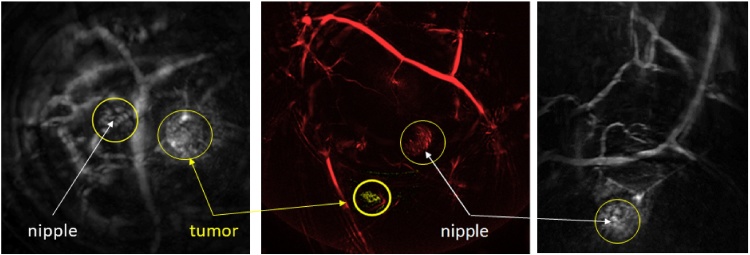
From the LOUISA 3-D hemispherical synthetic aperture imager: Maximum intensity projections of a full breast image from the LOUISA-3-D system: coronal x-y (left), sagittal x-z (center) and y-z (right) demonstrate blood vessels, microvessel-filled breast nipple and a small tumor. (Reproduced with permission of the authors and publisher.)

The authors reported on one patient result of the pilot study. The patient had a 3.5 mm lesion and a PA scan was made with 757 nm. Blood vessels and the tumor were visible in 2 out of 3 projections of the maximal intensity projections of the breast (Fig. 17).

Since only one wavelength was used, blood oxygen saturation of the blood vessels and the tumor was not possible. Further it is mentioned that the dignity of the small tumor was not conclusively ascertained. This result is thus claimed as a demonstration of the system's sensitivity, but not specificity.

A volunteer measurement is also described in the work, where two wavelengths were used allowing oxygen saturation analysis thereby allowing a discrimination between veins with reduced oxygen saturation and arteries with high oxygenation.

### 3.10 The Kyoto-Canon-Optosonics hemispherical system

#### 3.10.1 Instrumentation

This instrument referred to by the authors [56] as the PAM-03 system (for PAM-01 and PAM-02 see above Kyoto-Canon planar system), was developed by Canon Inc. (Japan) in collaboration with Optosonics Inc (USA), as a modification to the hemispherical geometry reported by the group of Kruger [92], [55], [54], [93].

*The ultrasound detector*. The US detector array is composed of 512 detectors distributed on the inner surface of a hemispherical shell with diameter 254 mm. Individual detectors are 3 mm in diameter made of piezoelectric zirconate (PZT), with a central frequency of 2 MHz.

*The patient-instrument interface*. The patient lies prone on a bed with her breast held within a cup made of a thin optically and acoustically transparent polyethylene terephthalate (PETG) membrane. The cup which is a hemispherical in shape (240 mm diameter, 38 mm depth) hangs into a chamber communicating with the imaging bowl formed by the hemispherical array beneath the bed. The extension chamber and bowl are filled with reverse osmosis water, and the cup carries a small amount of clean water ensuring acoustic coupling between detectors and breast. The water is maintained at body temperature during the measurements. The entire imager including the laser (see further) can be scanned with the breast cup kept steady.

*The laser and light delivery system*. The breast cup is illuminated from beneath along the rotational axis of the array. A Q-switched Alexandrite laser is the source providing approximately 200 mJ/pulse at 10 Hz, at selectable wavelengths of 755 and 795 nm. The beam is diverged to a diameter of 60 mm on the cup surface to yield a radiant exposure of less than 10 mJ cm^−2^ at the breast surface.

*The measurement protocol*. The FOV on the breast is determined by the opening angle of the US detectors and the distance of the array to the breast cup. The diameter of the light beam has been chosen to match this area. To improve the FOV, the bowl, extension and laser illumination is translated in the horizontal plane as one unit to trace out a spiral pattern. At each position the data from the array is acquired in parallel. The translation is implemented to have a higher density of positions towards the center of the spiral pattern, in order to improve image quality at the center of the breast cup corresponding to deep tissue. Depending on the number of positions the FOV improves up to 200 mm diameter. The total measurement time per breast using 2 wavelengths at the highest FOV is approximately 4 min [56].

*Signal processing and analysis*. Image reconstruction is based on a universal back-projection algorithm [78]. assigning different sound speeds for the water coupling and for the breast. The spatial resolution of the system measured by extracting the FWHM of the reconstruction of a spherical absorber (0.3 mm diameter) is reported as Δ_*x*_ = Δ_*y*_ = 0.57 mm; Δ_*z*_ = 0.37 mm.

Under the assumption of homogeneous background absorption (*μ*_*a*,*b*_) and reduced scattering ($\mu_{s,b}^{\prime }$), the fluence distribution at the PA pressure locations was approximated by the diffusion approximation. It appears [77] that values of *μ*_*a*,*b*_ and $\mu_{s,b}^{\prime }$ per breast were estimated using time-resolved spectroscopy, applied in breast volumes far from lesion locations. From the optical absorption factor at the PA sources ${\tilde{\mu }}_{a}$ calculated in this way for the two wavelengths, a blood oxygen saturation index (S-factor) was calculated, knowing the molar extinction coefficients of oxy- and deoxy-Hb from the literature. A S-factor image was developed using intensity assigned to the ${\tilde{\mu }}_{a}$ at 795 nm as being proportional to total HB concentration, and with a color hue assigned to the S-factor value.

Motion correction in 3-D was applied to images between laser pulses, and as well as to images between wavelengths. For the former, a voxel-based method using the sum-of-squared distance as a similarity measure between was applied to images reconstructed between laser pulses. For the correction between wavelengths, a free-form deformation method for solid objects was employed [94], to deform the breast shape of the 755 nm image towards that of 795 nm image. Subcutaneous tissue voxels were removed, and a cloth-simulation approach [95] applied to retain surface information. For image interpretation of deeper-lying regions, volume data was removed from surface to an arbitrary depth.

#### 3.10.2 Patient studies

Twenty-five patients, of which 22 were malignant cases, were included in the study. The affected breast was scanned first, followed by the contralateral breast, at both wavelengths. Images were assessed by 5 readers, who scored 5 questions pertaining to visibility and nature of vascular signals around and in the tumor, and other intensities in the tumor.

The following are the most important findings:

(1) *PA breast imaging shows blood vessels superior to MR*

**Fig. 18:**
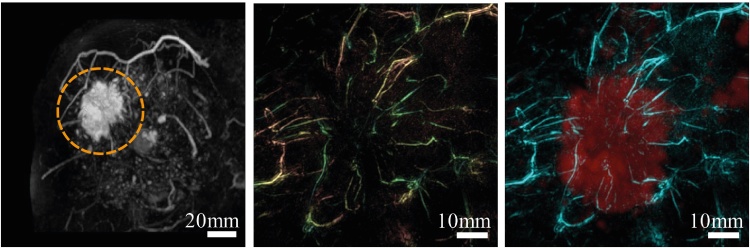
Using the Kyoto-Canon-Optosonics hemispherical imager: the case of a 40-year-old woman with invasive breast cancer, with a 47 mm diameter tumor. First column from left: MR image with lesion marked with red circle. Second column: PA image after eliminating the signals from subcutaneous blood vessels within a depth of 4 mm. Third column: Fusion images of PA (cyan) and MR (red). The PA images demonstrate that tumor associated vasculature converges toward the center of the tumor, tapering drastically at the tumor edge and near the center, where they disappear. (Reproduced with permission of the authors and publisher.)

Using the hemispherical array it is possible to obtain 3-D high-resolution visualization of fine vasculature with higher details than on standard contrast-enhanced MRI (Fig. 18).

(2) *Differences in PA signals in and around tumor*

Centripetal vasculature directed toward the tumor was identified as shown in Fig. 18. There was also disruption or rapid narrowing of vessels at the tumor boundary. Often vascular-like linear signals and spotty signals inside tumors were also seen. Further, there appeared significant differences in peritumoral vascular density in most IBC cases in comparison with healthy contralateral breasts.

(3) *Differences in PA appearances between IBC and DCIS*

Centripetal vasculature signals directed toward the tumor were detected in 61% of IBC cases, compared with 35% of DCIS cases.

(4) *Changes in the PA appearances of tumor-vascularity before and after neoadjuvant therapies*

Fine intratumoral vasculature became visible in the PA images after chemotherapy. Additionally changes in S-factor around the lesion, could be observed after chemotherapy. On the other hand no significant change in tumor size could be detected by US. This is a significant result suggesting that normalization of tumor vasculature may be driven by chemotherapy.

(5) *PA breast imaging shows blood vessels superior to MR*

PA imaging of the breast deeper than 30 mm was problematic. This may be due to loss of image quality and accuracy of S-factor estimation, due to motion artifacts despite motion correction processing. It is important to speed-up data acquisition and improve reconstruction to extend the high resolution imaging to deeper tissues.

## 4 Discussion and outlook

It is noteworthy that there is a wide variety of system geometries in PA breast imaging, unmatched in x-ray mammography, breast MR and breast US where largely the same implementations are in use per modality (see Table 1). The geometry and features of the US detection aperture dictate the PA imager's geometry, the patient-instrument interface, its performance and potential applications. Generally, a division may be identified in the implementations – hand-held probe imagers, and dedicated breast imagers. The first category comprises imagers based on hand-held US detector probes, linear or curved, which contact the breast and are used to manually scan regions of interest with the patient supine as in breast echography (Sections 3.1–3.3). Most of the devices also perform US imaging. The imagers have the external appearances of conventional medical US imagers, with the addition of light-delivery systems and lasers. These devices are also real-time capable. Like in their conventional US imaging counterparts, the hand-held probes used have the form-factor to be applied to other organs as well, making the instruments versatile.

**Table 1:** Overview of systems with their technical specifications and imaging performances.

System	Technical			Clinical			
	Detector properties	Laser properties	Imaging performance	Study name/year	Number of lesions	Lesion type	Detected/total
*Handheld*							
Seno Medical Imagio linear	*f*_0_ = 7.5 MHz BW = 0.1–10 MHz 128 elements	755, 1064 nm <100 ns pulses	±35 mm depth	Pioneer 2017	1079 678	Benign Malignant	43% specificity 96% sensitivity
				Maestro 2018	146 benign	FA Papilloma Phyllodes Microcyst Other	65/75 3/6 1/1 13/16 36/48
					67 malignant	IDC ILC Papilloma DCIS Other	45/47 7/8 3/3 2/2 7/7
Technical University of Munich (TUM) curvilinear	*f*_0_ = 5 MHz 256 elements 174° span	680-980 nm <10 ns pulses	–	2017	10	NS ILC	?/8 ?/2
University Hospital Münster (UKM) curvilinear	*f*_0_ = 3 MHz BW = 56% 256 elements 125° span	680–930 nm 30 mJ/pulse	40 mm depth 0.25 mm resolution 40 × 40 m^2^ FOV	2017	7	IBC DCIS	5/5 0/2

*Planar*							
Kyoto-Canon Planar system 1	*f*_0_ = 1 MHz BW = 80% 345 elements	756, 797 nm 85 + 60 mJ/pulse 7 ns pulses	50 mm depth <1 mm resolution 30 × 46 mm^2^ FOV	2012	27	IBC DCIS Phyllodes	15/21 5/5 0/1
				2015	39	IBC DCIS	24/33 5/6
Twente PAM planar system	*f*_0_ = 1 MHz BW = 130% 590 PVDF elements MDP = 80 Pa	1064 nm 60 mJ/pulse 10 ns pulses	35 mm depth <2 mm resolution 85 × 90 mm^2^ FOV	2007	6	IDC Cyst	4/5 0/1
				2012	12	IDC ILC IDC + ILC Cyst	7/7 1/1 2/2 0/2
				2015	36	IDC ILC LCIS FA Cyst	24/25 3/3 1/1 2/2 5/5

*Ring*							
LOIS-64 curved	*f*_0_ = 1 MHz BW = 0.1–2.5 MHz 64 elements 1.66 mV/Pa	757, 1064 nm 750 mJ/pulse 75 ns pulse	50 mm depth 0.5 mm in plane resolution	2009	27	Carcinomas Papilloma Cyst FA Fibrosis	17/25 1/1 1/3 2/3 1/2
University of Florida ring	0.34–1.48 MHz 64 elements	733, 775, 808 nm 78 mJ/pulse 8–25 ns pulse	0.5 mm resolution	2015	6	IDC DCIS	4/4 2/2
Caltech Single-Breath-Hold ring	*f*_0_ = 2.25 MHz BW = >95% 512 elements 360° span	1064 nm	40 mm depth 0.258 mm in plane resolution 5.6 mm elevation	2018	9	IDC ILC DCIS Fibrosis	3/3 1/1 3/3 2/2

*Hemispherical*							
Louisa-3D synthetic aperture hemispherical imager	*f*_0_ = 0.05–6 MHz 96 elements 90° span 0.012 mV/Pa	757, 797 nm	0.3 mm resolution	2018	1	Unknown	1/1
Kyoto-Canon hemispherical imager	*f*_0_ = 2 MHz BW = 70% 512 elements	755, 795 nm 200 mJ/pulse	27 mm depth 0.4 mm resolution	2017	22	IBC (ILC and IDC) DCIS	12/17 3/5

Abbreviations: FOV = field of view, IBC = invasive breast cancer, IDC = infiltrating ductal carcinoma, ILC = infiltrating lobular carcinoma, FA = fibroadenoma, DCIS = ductal carcinoma in situ, NS = non-specific cancer.

On the other hand, several implementations are dedicated for breast imaging. All of these encompass and image the breast completely or to a large extent, usually via dedicated water coupling. The detectors are configured as planar, curved, ring or hemispherical arrays. (Sections 3.4–3.10) The patient lies prone on an examination table or bed, with her breast pendant or supported in the imager.

Generally the hand-held probe imagers are being developed with ambitions for applications in diagnosis, while many of the dedicated devices have explicit or implicit aspirations for application in screening. This leads to the question about the role which photoacoustics will play in the breast imaging paradigm, which we will touch upon further.

At this juncture, photoacoustics is a technique that has achieved the capability of making exquisite breast images of predominantly blood vessel distributions, and showing areas where tumors are present. To elevate PA imaging from being merely the technique with potential for clinical application into a modality with strong impact in patient care, we believe that these issues require attention in the coming period:

### 4.1 Photoacoustic appearances of diseased and healthy breast

The contrast for PA imaging comes from optical absorption, the strongest biochrome in tissue being hemoglobin (Hb) in blood. It is widely accepted that sustained vascular activity is a hallmark of cancer [24], and also that tumor vasculature is different from normal vasculature in biological behavior as well as in architecture [25]. This has been the rationale behind using photoacoustics for the imaging of breast cancer. Further, progressive tumor tissues can become hypoxic and necrotic because of rapid cell proliferation and insufficient blood supply. Imaging oxygen saturation in tumor tissue with photoacoustics, relying on the differences in optical absorption of Hb and oxy-hemoglobin (HbO_2_), could be an indicator of tissue metabolism. Dysregulated metabolism is also considered to be a hallmark of cancer [24]. Malignancies or more aggressive phenotypes possessing higher metabolism may demonstrate lower oxygen saturations [96]. Some work has shown that this may be the case *in vivo* using DOT (see for example Refs. [97], [98], the latter on ex vivo samples), and this should be confirmed with appropriate studies with PA imaging on large cohorts with appropriate validation.

Various recent studies discussed [36], [39], [56], [52] have shown high peripheral blood vessel densities and higher total blood volumes in cancer. However, a new finding [36] is that PA can reveal the stratified organization of breast tissue. This work made possible with the use of high resolution, high element-density detectors with a large number of excitation wavelengths, demonstrates patterns of Hb, HbO_2_, fat and water in composite images which match with the stratified anatomy of the healthy breast (Fig. (6)). Significantly, the authors found that this organization is slightly to markedly disrupted in the disease condition compared to healthy breast, due to surrounding tissue response to tumor infiltration (Fig. (7)). This is a new way of assessing and analyzing tissue associated with lesions not offered by other clinical imaging modalities, which potentially could provide information supporting detection or diagnosis. This should be further investigated on larger patient numbers with appropriate validation.

While there is no uniformity in technical choices, as we have seen in this review, the appearances of tumors and surrounding tissue which have been reported has also been varied. For example, at tumors sites some work has shown the clustering of hot-spots that may produce recognizable features such as mass-like and even ring-shaped (Figs. 11, 15 and 7), [71], [36], [84] others have shown only peripheral blood vessels which has been interpreted as feeding blood vessels (Figs. 12 and 18) [48], [56]. These have been shown to have higher densities and demonstrate irregular sprouting and branching [80]. The reasons for the differing appearances are likely due to differing detection apertures, and due to choices made for the characteristics of the US detectors, as well as the form of illumination zones and laser wavelengths used. A reason could also be a pathophysiological one, in that intra- or peri-tumoral vascularity may not be highly reliable as markers for the disease, and that the vessel density may not always be high enough to provide the optical absorption contrast to visualize lesions, especially in the presence of larger superficial blood vessels. This will have to investigated in detail.

Much is still required to be learnt from *in vivo* imaging studies, however, it is equally important at this juncture, to return to basics and perform *in silico* imaging studies. This would require accurate simulations of light propagation [99], pressure generation, acoustic propagation [100], and detection with various instrumental characteristics of the imagers, in highly accurate numerical phantoms simulating the breast [58] and various lesions. This will be the way to understand the appearances of the breast anatomy and various tumors, and may also guide the choice of various imager characteristics for different applications.

#### 4.1.1 PA imaging and contrast agents

As mentioned, the intrinsic contrast of breast cancer in PA imaging is from tumor vascularity, which provides an optical absorption contrast for signal generation. It is as yet unclear if vascularity is a reliable indicator of breast disease, and powered clinical studies with rigorous validation are required. If the pathophysiology does not provide a reliable imaging contrast for detection and diagnosis of breast cancer, then the use of contrast agents may be required.

Diffuse optical tomography provides insights into the use of optical contrast agents, especially with the use of the FDA approved blood flow agent Indocyanine Green (ICG) [101], [102]. This fluorescent dye has an absorption peak at 780 nm in water medium, with a poor radiative de-excitation efficiency making for a useful photoacoustic contrast agent. Bolus administration of ICG in the blood stream results in binding of the dye to blood proteins revealing in DOT what may be interpreted as vascularization maps. This approach may be favorable to achieve localization and delineation of tumors using photoacoustics, if sufficient optical absorption contrast can be developed. In DOT it has also been observed that malignancies may possess slower kinetics of uptake and outflow of ICG compared to healthy tissue or benign lesions [102], [103]. This feature may also be exploited by photoacoustics providing further parameters for diagnosis.

As yet there have been no reports using ICG in breast cancer with photoacoustic imaging. The well-known FDA approved dye methylene blue (MB), widely used as guidance to the eye in sentinel node biopsy, has been used for photoacoustic discrimination of the sentinel node from downstream nodes [31]. While MB can provide a niche role in tumor “node” metastasis (TNM) breast cancer staging [104], its use for detection and diagnosis of the disease is considerably less likely due to its absorption peak lying at 664 nm where light penetration is lower than at the longer NIR wavelengths.

While molecular imaging with targeted contrast is much sought after, and elegant and convincing studies have been performed using targeted agents including nanoparticles, this has been restricted to preclinical models. A few excellent reviews consolidate the work in area, and the outlook of such agents for human use [105], [106].

### 4.2 The penetration depth of light

In clinical imaging modalities, the penetration into breast of the probing energy fields used – X-rays, ultrasound or magnetic fields – is excellent to good. In PA imaging, the light penetration to excite ultrasound is relatively limited, with the fluence in breast tissue reducing by roughly 1 dB per cm of propagation in the far-red and NIR wavelength regimes, due to absorption and multiple scattering of photons. The important absorbers in breast tissue at these wavelengths are melanin, H_2_O, lipids, Hb and HbO_2_. Melanin due to its high localization in a thin superficial layer likely has a negligible effect in full breast tissue volumes, while H_2_O and lipids though widespread in breast tissue have low absorptions in the NIR wavelength regimes. Hb has a high absorption, and is also widely distributed in the tissue with volume fractions between 1% and 5% and is the dominant endogenous chromophore. Light penetration till around 1100 nm thus inversely follows Hb absorption to a large extent. Including multiple scattering of light in the diffusive regime, in a one-dimensional approximation, the incident fluence diminishes as *ϕ*(*r*) = *ϕ*_0_*e*^−*μ*_eff_*r*^ where *μ*_*eff*_ is an effective attenuation given by $\mu_{\mathrm{eff}}=\sqrt{3\mu_{a}(\mu_{a}+\mu_{s}^{\prime })}$, where *μ*_*a*_ and $\mu_{s}^{\prime }$ are respectively the optical absorption coefficient and reduced scatter coefficient. The mean penetration depth (*δ*), the depth at which the fluence in tissue falls to *e*^−1^ of its initial value, for a simplified model breast tissue (comprising 1% blood with 65% oxygenation, 35% lipid, and 35% water by volume) shows that the penetration depth reaches around 10 mm in the wavelength range 700–1100 nm [57].

This behavior has the following important implications:

(i) Imaging depths in general for PA breast imaging may be limited.
(ii) Due to the fluence *ϕ*(*r*) dropping considerably in the large amounts of breast tissue, the optical energy absorbed *H*(*r*) = *μ*_*a*_(*r*)*ϕ*(*r*) and pressure *p*_0_(*r*) = Γ*H*(*r*), produced from deep inside the breast is small, calling for the requirement of highly sensitive US detectors. A further requirement is for a high dynamic range in the US detector-digitizer measurement chain, since the signals produced deep inside tissue are several dB lower than at the surface. Otherwise signals from superficial structures will be saturated, or the signals from deep inside will be lost in the noise floor.
(iii) Due to the spatially variant fluence *ϕ*(*r*) the PA signal at depth is not directly proportional to *μ*_*a*_(*r*) but is dependent on *μ*_*a*_(*r*)*ϕ*(*r*), with *ϕ*(*r*) itself depending on *μ*_*a*_ (*μ*_*s*_ and the anisotropy *g*). This constitutes a non-linear problem, that makes the accurate estimation of *μ*_*a*_(*r*) and the chromophore concentrations difficult.

Regarding (i) the breast lends itself to illumination from all around, when it is pendant, in a dedicated breast imaging configuration with a hemispherical US detection aperture. If in such a geometry, the light delivery is so arranged as to get a homogeneous light fluence distribution on the entire breast surface, full-breast imaging is possible. This is a bigger challenge in hand-held geometry. However, in order to be able to interrogate the entire breast and extract meaningful data, the problems described in (ii) and (iii) need to be addressed, namely that US detection is made highly sensitive, and that the fluence in all locations is measured or estimated in an accurate way.

As mentioned above while skin color is likely to have a marginal effect in influencing light penetration, in order to improve our understanding of the applicability of the photoacoustic method in breast imaging universally, it will be useful to record skin pigmentation of subjects. The 6-point Fitzpatrick scale [107] which classifies skin types based on response to UV but often used to describing skin color can be used. Alternatively, an objective measure of the skin color using diffuse reflectance spectroscopy (DRS) [108], [109] may be employed.

### 4.3 Quantitative Multispectral imaging and testing on advanced phantoms

In addition to the visualization of lesions from tumor associated vascularity, the quantitative estimation of chromophore concentrations in breast tissue is important. This latter information is necessary to ascertain the pathophysiological status of the tissue, as the relative presence and distributions of the chromophores are different in health as in disease. Quantitative imaging is possible by exploiting the specific spectral signatures of the chromospheres within the far-red and NIR regions, necessitating the use of multiple wavelengths. While information regarding distributions of lipids and H_2_O may provide important landmarks in the breast and may be altered in breast cancer [36], the quantification of Hb and HbO_2_ and thereby estimation of SO_2_ is of main interest, as it is expected to provide an assessment of tissue metabolism, which is known to be dysregulated in cancer [25], [24]. An accurate estimation of SO_2_(*r*) is non-trivial for the reasons explained, namely the unknown spatial distribution of light fluence. Further, the optical properties *μ*_*a*_(*r*) and *μ*_*s*_(*r*) change with wavelength, which means that the light fluence changes with wavelength further compounding the problem.

In a zeroth approximation it is assumed that fluence changes at an ROI for various wavelengths are marginal, and the values of PA image voxels are directly used to estimate the relative presence of Hb and HbO_2_. However, the method is inaccurate, and theoretical models such as the radiative transfer equation (RTE) and the Diffusion Approximation (DA) of the RTE, can be employed as surveyed in detail in Refs. [110], [111]. Experimental strategies use additional measurements typically diffuse optical transmission and reflectance, to estimate the *μ*_*s*_ and *μ*_*a*_, and thereby fluence. This has also recently been demonstrated in a hand-held geometry [112]. A recent technique also uses additional acousto-optic measurements to compensate for the fluence [113]. Yet while many approaches have shown potential, these have been demonstrated only numerically or in simple laboratory experiments. A stringent test of these approaches will be when they are applied to situations which replicate the practice as closely as possible with non-ideal detectors, inhomogeneous illuminations, and uncertainties and noise in measurements. This calls for acquiring data sets with the developed instrumentation from:

(i) well-characterized phantoms that simulate the complex acoustic and optical spectral properties of healthy and diseased breast tissue to the level of sophistication required [114], [115], [116], [117], [118], [119];
(ii) carefully-controlled laboratory experiments that are able to locally control the optical properties to mimic SO_2_ at the simulated disease site, with appropriate validation tools [120].

Only after satisfactory studies in such settings, will it be possible to identify the most robust, accurate and practical approaches that have a high probability of success in performing quantitative imaging in human subjects.

Imaging SO_2_ (and chromophore concentrations) in 3-D with sufficient accuracy, from multi-wavelength measurements, remains one of the key challenges in the field. A computationally feasible or experimentally practical solution will enable PA breast imaging to achieve its true potential since with this comprehensive assessment of breast tissue will be possible. This could provide robust criteria to discriminate between benign, pre-invasive and malignant lesions, and perhaps even help stratify tumors based on aggressiveness enabling a more accurate detection, diagnosis or other role as discussed further.

### 4.4 Hybrid imaging

Photoacoustic breast imaging is still in its infancy, with not more than 10–12 research prototype imaging instruments and a handful of ongoing clinical studies, most of which are demonstration studies. The variation in instrumentation is also unprecedented which may predominantly be responsible for the variation in appearances of gross image features, and specifically in appearances of lesions. The method is also intrinsically hybrid involving electromagnetic excitation using light, and measurement of the resulting mechanical energy as ultrasound. For a good understanding of the information acquired using the method, we should be able to understand (and quantify) in all situations light delivery characteristics, light interactions in tissue, the US generation process, US interaction in tissue, and US measurement characteristics. This is challenging. The net result is that PA images are complex, and interpretation of these is difficult.

US on the other hand is ubiquitous in the breast clinic and images are well interpreted. Breast US is able to delineate the skin, the nipple, fat, glandular tissue and various lesions [3]. Integrated US and PA imaging in a hybrid imager, would be the means to place the information related to vascularity from PA, in the context of well-known anatomic landmarks in the breast from US. From the viewpoint of US, the method is used primarily for cyst/solid mass discrimination [3], and is also being investigated to discriminate benign solid tumors from malignant masses [121]. In such a multi-modal US-PA imager, information regarding tumor vascularity could help US by providing an additional feature to improve diagnosis within solid masses. Real-time multi-modal PA-US systems will see application in guidance of biopsy as well.

From an implementation standpoint, the US detection hardware required for both techniques can be shared [40]. In hand-held systems, this is implemented using off-the-shelf probes, standard electronics, high-voltage electrical circuitry and image reconstruction software used in conventional US imagers. For dedicated breast systems, the US transducers and driving electronics will have to be developed specifically for the purpose. Thus the development of hybrid PA-US is crucial for improved interpretation of PA images, and also provides complementary and synergetic information between the two methods, while reducing the need for separate multiple imaging as seen in the diagnosis of breast cancer. This considerably improves the opportunities, and chances of success in the clinic.

The question arises whether PA could be used as an adjunct to X-ray mammography, in a single implementation as a hybrid instrument. The requirement for PA imaging may be to improve the specificity of X-ray imaging alone in a screening setting (see Section 4.5.1), with the addition of a spectroscopic and thus functional imaging variant. Optical mammography has been developed as a combination with a standard mammographic or digital breast tomosynthesis (DBT) system, where the optical sources and detector probes can be readily attached and detached from the transparent source cassette and compression paddle (see for example Refs. [122], [123]). For the case of PA imaging in a planar geometry, the optical illumination scheme can be similar to the optical mammography system, and poses no fundamental problem with the transparent compression paddle [124]. Acoustic detection is more challenging because of the requirement for an acoustic coupling medium with low acoustic attenuation between the detector and breast tissue, which would be impeded by the source cassette which while being optically transparent is unlikely to be acoustically transparent. Ultrasound detection from the sides where access to the breast is available may be possible, to provide a dark-field detection scheme.

### 4.5 Applications in the breast cancer management model

Imaging activities related to breast cancer begin with screening for the disease in asymptomatic women, and go further at all the phases through diagnosis, staging, guiding biopsy, monitoring neoadjuvant therapies, guiding surgery and surveillance. Most imaging technologies are developed specifically for two distinct roles:

1. to screen: identify and locate abnormal tissues in the breast for further examination;
2. to diagnose: characterize the abnormality and to enable informed decision on treatment course.

An ideal technology will accomplish both goals, but in the real world this is not possible yet. Technologies are thus optimized for one role at a time, and then depending on relative advantages and disadvantages, are chosen for application in the other phases in the breast management model. In this section we will discuss only the opportunities and challenges for PA breast imaging in the roles of screening and diagnosis.

#### 4.5.1 Screening for breast cancer

Early detection of breast cancer is believed to reduce breast cancer mortality. The only imaging method that has been validated for screening is X-ray mammography. Studies have proven that X-ray mammography finds early stage breast cancer, but the findings on whether disease-specific mortality has been reduced are inconclusive [125], [126], [127]. If mortality is not reducing, then this suggests that X-ray mammography may be finding more less-lethal and slower progressing cancers, as interval cancers are still being reported. Further, specificity is low and it has been estimated that ≤50% of abnormal screening mammograms will prove to be negative [128]. Most patients referred to a specialized breast cancer care center after a positive screening will have benign disease [3]. Further, the performance of X-ray imaging in the dense breast is generally poor. Other drawbacks are that X-ray mammography requires painful breast compression to be applied, and that X-rays constitute ionizing radiation.

It must be mentioned that the requirements of a new imaging technology for breast cancer screening are extraordinarily stringent, compared with other roles. The instrumentation has to be high throughput, simple to operate for staff, inexpensive and with a low burden to subject. The technology should be disposed towards high sensitivity (true positive rate) so as to not to miss potential disease to a level as to being higher than 90–95% as in X-ray mammography. The specificity should be higher than that of X-ray mammography.

While the above requirements are in an operational sense, the ultimate objective of screening, in terms of outcomes is to reduce the complications and mortality rate of breast cancer among the women screened, with low harms of overdiagnosis and/or overtreatment. Since population-wide screening is a public-health consideration that requires a broad societal, medical-professional, financial, regulatory and political acceptance, evidence of benefits outweighing the harms through randomized control trials is crucial. Further, since the method is intended to be used on large numbers of asymptomatic women to identify a small number of potential cases, large clinical trials are required.

With quantitative PA imaging's ability to image vascular patterns and oxygen status, and PA-US multi-modal imager's capability to image morphology, the technique definitely has potential to improve screening especially with the promise of high specificity. However, enthusiasm to develop, test and apply the method in screening should be tempered since the tasks for making inroads into this role are Herculean. It is good to keep in mind that breast screening is one of the most controversial medical procedures, and any other new technique will be highly scrutinized at multiple levels.

*Photoacoustic imaging and the dense breast*: There has been as yet no study explicitly addressing any benefits in tumor visualization in the dense breast. Two small studies [70], [73] on enriched cohorts of 12 and 31 patients respectively, which maintained data of BIRADS breast density classifications from mammograms, made the observation that tumor contrast from photoacoustic imaging remained stable irrespective of breast density. Other small studies have also reported similar results, for example, Ref. [129].

From the point of view of light interactions with tissue in the NIR regime, it is not readily clear why this has been observed. The dense breast is characterized by higher proportion of fibroglandular to fat tissue compared with the ‘normal’ breast. This is expected to be characterized by greater vascular densities, water content and metabolic activity. DOT spectroscopic studies pointing to higher HbT, H_2_O, lower deoxy-Hb and generally higher scattering. This would play a role in reducing penetration depths and reducing tumor contrast. This effect has however not been observed in DOT. One pilot study looking specifically at tumors in dense breast tissue, concluded that the high spectral content of diffuse optical spectroscopy DOS imaging measurements allowed the visualization, detection and characterization of malignancies in dense breast regions [130].

#### 4.5.2 Diagnosis in breast cancer

Women who are screened positively or who exhibit symptoms of breast disease require diagnostic workup to ascertain whether or not they actually have the disease. The goal of diagnosis is thus to establish whether a screen-detected or otherwise found abnormality is a cancer or a benign process. This differentiation is performed by integrating information from a clinical examination, imaging and biopsy. With breast cancer being a complex group of diseases, and no one imaging method having characteristics that allow it to be used in isolation, a multi-modal, layered approach is used in the diagnostic trajectory. Imaging is performed using diagnostic X-ray imaging and US imaging, with MRI being used with increasing frequency. As mentioned often, all of the present imaging technologies have limitations. The requirements of imaging in diagnosis are highly stringent but less so than in screening. From an outcome viewpoint the requirement of imaging in diagnosis is to provide a definite confirmation of the dignity of a lesion leading to a specific course of treatment. Narrower outcomes can also be sought, such as downgrading (or upgrading) suspiciousness of certain lesions that will result in biopsies avoided (or conducted) (Section 3.1).

The imaging method works with relatively low numbers of women with a higher prevalence of breast disease. From an operational viewpoint, the imaging procedure can take more time, may entail higher burden to the patient, may be more invasive and may be more expensive. There is a higher weightage to accuracy and precision than to patient acceptability. The method needs generally to be chosen towards high specificity.

With the use of quantitative image reconstruction of multi-wavelength PA data functional information of tumor vasculature and oxygen saturation can be ascertained. This is expected to be highly specific to cancer. From the US part anatomic features of the lesion can be demonstrated. Such a combination which leads to a comprehensive assessment of the lesion can be powerful tool in the diagnostic trajectory.

Naturally scientific evidence for the efficacy and utility of PA imaging in diagnosis or in a niche role in the diagnostic trajectory will have to be obtained in well designed and well implemented clinical studies.

## 5 Concluding remarks

The road towards clinical translation is a long and expensive one, and is especially treacherous. A difficulty in evaluating a new imaging technology compared to a therapeutic one is that, unlike the latter, imaging does not provide a direct health outcome. Rather imaging generates information which can lead to a certain therapeutic decision out of several options. However this information is subject to interpretation, and this information is also not considered in isolation during the decision making process. Thus, the evaluation of such a technology boils down to an assessment of the value of imaging information in indirectly producing health outcomes [5]. This is complex, and calls for well-designed studies to produce evidence of benefit. However before such studies are undertaken, it is good to be aware of caveats in the field of PA imaging. One should be mindful of the high number of PA instrument sub-system technical parameters, their interplay, the wide variability of image reconstruction methods and the effect of all of these on imaging performance. One should also be mindful of the inherent complexity of the PA signals and the resulting challenges in interpretation of *in vivo* PA images. Further, the state-of-the-art PA imagers still require iterations in their development, testing and validation cycles before the biological capability of the method in imaging the breast in health and disease is fully understood.

In the last few years, the frontiers of photoacoustic breast imaging have been rapidly advanced, with the frequent appearance of new imaging instrumentation and several *in vivo* studies mostly, but not always, in the nature of technology demonstrations (Table 1). These studies re-confirm the potential and promise of the photoacoustic method in detection and diagnosis of breast cancer. Taken together with the challenges above, this makes for a fascinating passage in the near-future towards translation; it is an exciting time to be in the field of PA breast imaging [72].

The key to success will be instrumentation research, technology development and clinical assessment in an academia-hospital-industry nexus, with the role of industry in engineering, manufacture, and planned commercialization quickly developing to a crucial one. Further, when evidence of benefit of the method becomes clearer in the future, other stakeholders, such as entities responsible for the regulatory and approval process for medical devices, for insurance coverage, and for reimbursements, will become increasingly important.

## Conflict of interest

The authors declare no conflicts of interest.

## Footnotes

1 MSOT – multispectral optoacoustic tomography.

2 Here we use MSOT and multispectral photoacoustic imaging to mean the same.
